# Medical cannabis use in Australia: consumer experiences from the online cannabis as medicine survey 2020 (CAMS-20)

**DOI:** 10.1186/s12954-022-00666-w

**Published:** 2022-07-30

**Authors:** Nicholas Lintzeris, Llewellyn Mills, Sarah V. Abelev, Anastasia Suraev, Jonathon C. Arnold, Iain S. McGregor

**Affiliations:** 1 Drug and Alcohol Services, South East Sydney Local Health District, Sydney, NSW Australia; 2 Department of Addiction Medicine, Faculty Medicine and Health, University of Sydney, 591 South Dowling Street, Surry Hills, NSW 2010 Australia; 3 Lambert Initiative for Cannabinoid Therapeutics, The University of Sydney, Sydney, NSW Australia; 4 Faculty of Science, School of Psychology, The University of Sydney, Sydney, NSW Australia; 5 Brain and Mind Centre, The University of Sydney, Sydney, NSW Australia; 6 Department of Pharmacology, Sydney Pharmacy School, Faculty of Medicine and Health, University of Sydney, Sydney, Australia

**Keywords:** Cannabis, Medical cannabis, Medicinal cannabis, Consumer survey

## Abstract

**Background:**

Australia has had a framework for legal medicinal cannabis since 2016, yet prior online surveys in 2016 and 2018 indicated that most consumers continued to use illicit medical cannabis products. Regulatory data indicate an increase in the prescription of medicinal cannabis since 2019, and this survey examines consumer experiences of prescribed and illicit medical cannabis (MC) use in Australia.

**Methods:**

A cross-sectional anonymous online survey was administered September 2020 to January 2021. Recruitment via social media, professional and consumer forums, and medical practices. Participant eligibility: ≥ 18 years; used a cannabis product for self-identified medical reason(s) in the past year, and resident in Australia. Outcome measures included *c*onsumer characteristics, conditions treated, source and patterns of MC use, and perspectives on accessing MC.

**Results:**

Of the 1600 participants (mean age 46.4 ± 14.3 years, 53% male), 62.4% (*n* = 999) reported using only illicit and 37.6% (*n* = 601) used prescribed MC in the past year. MC was used on a median of 28 (IQR: 12, 28) of the past 28 days and cost $AUD 74 ± 72 weekly (median = $40, IQR: $7, $100). Prescribed participants were more likely to treat pain conditions than those using illicit MC (52% v 40%, OR = 1.7, 1.3–2.1) and less likely to treat sleep conditions (6% v 11%, OR = 0.5, 0.3–0.8), with mental health conditions also a common indication in both groups (26%, 31%). Prescribed MC was consumed predominately by oral routes (72%), whereas illicit MC was most commonly smoked (41%). Prescribed MC was ‘mainly THC’ (26%), ‘equal THC/CBD’ (40%), ‘mainly CBD’ (31%) and ‘uncertain’ (3%), while 34% of those using illicit MC were ‘uncertain’ of the cannabinoid profile. Cost and difficulties finding medical practitioners to prescribe remain significant barriers to accessing prescribed MC, and few (10.8%) described the existing model for accessing prescribed MC as ‘straightforward or easy’.

**Conclusions:**

There has been a notable shift from illicit to prescribed MC by many consumers compared to prior surveys. Consumers using prescribed MC reported a range of advantages compared to illicit MC, including safer routes of administration, and greater certainty regarding access and composition of products.

**Supplementary Information:**

The online version contains supplementary material available at 10.1186/s12954-022-00666-w.

## Introduction

There is a global trend towards increasing use of cannabis-based products for therapeutic or medical reasons. A number of countries across North America, Europe, Asia and Oceania have introduced regulatory systems that allow consumers to access legal medical cannabis products, although there is considerable variation between countries as to the types of products available (e.g. forms and routes of administration, cannabinoid composition and quality control requirements), how these are accessed in the health system (e.g. prescribed by medical practitioners and dispensed at pharmacies, or available over-the-counter without prescriptions at special dispensaries), and with variation as to the range of conditions that can be treated with cannabis products [1].

In Australia, a range of cannabis-based products have been able to be prescribed by any medical practitioner (without special credentialing) since November 2016 as unregistered medicines using the compassionate access regulatory pathways (Special Access (SAS) and Authorised Prescriber Schemes [2]). There are over 200 such products available at the time of writing [3], with a range of cannabinoid (tetrahydrocannabinol (THC) and cannabidiol (CBD)) profiles and routes of administration (mainly oral solutions and plant matter for vaporisation), and which comply with Good Manufacturing Practice standards. Cannabis-based medicines can be dispensed by any community pharmacy and can be delivered by mail or courier from a pharmacy to patients with a valid prescription. Australian clinical guidance, educational materials and professional development programmes are available to assist clinicians to deliver medicinal cannabis treatment [4–8]. These guidelines highlight the evidence available for a range of conditions (e.g. chronic pain, palliative care, chemotherapy, epilepsy); however, in practice, doctors may prescribe for any clinical indication as long as they can justify prescribing to the regulating body based on the available evidence. A recent analysis of Australian regulatory data suggests the majority of patients are treated for the indications of pain, anxiety or sleep disorders, and that the majority of prescriptions involve THC-based medications rather than CBD-only products [9]. While prices vary between different products, at the time of writing, THC-predominant herb products often cost the consumer between $AUD 15–20 per gram (not dissimilar to prices reported by many consumers for illicit cannabis). CBD-predominant oral solutions are generally more expensive to the consumer, costing at least $AUD 0.67 per mg (i.e. approximately $AUD 26 for a daily oral dose of 400 mg).

It remains uncertain how the increased availability of prescribed medicinal cannabis has impacted overall patterns of medical cannabis use in the community. The 2019 National Drug Household Survey [10] reported that 11.7% of Australians (2.5 million people) aged ≥ 14 had used cannabis in the previous year, of whom 23.1% (~ 600,000 Australians) used cannabis for medical reasons: 6.8% for medical purposes only and 16.3% for both medical and non-medical reasons. Only 3.9% of respondents using cannabis for medical purposes obtained their cannabis by prescription. This is consistent with our earlier 2018–2019 online survey [9] in which only 2.4% of 1044 respondents indicated that they had accessed MC via prescription, with most using illicit cannabis. Both surveys hinted at demographic and clinical differences between people using prescribed versus illicit MC, but the small numbers of participants in the former category limited any meaningful analysis.

Since those surveys, Australian Therapeutics Good Administration regulatory data [11] demonstrate a marked increase in the uptake of prescribed MC, with the number of regulatory applications by doctors to prescribe medicinal cannabis products increasing from 231 in 2017, to 2560 in 2018, 25,160 in 2019, 57,710 in 2020 and 122,490 in 2021. This dramatic increase in regulatory applications has largely been accommodated by both general practitioners and medical specialists, with most Australians having access to government health insurance systems (Medicare) that partially subsidises the cost of medical consultations, although there is no reimbursement for medicinal cannabis products dispensed at pharmacies. There has also been the establishment of a number of private ‘cannabis clinics’ in recent years, which anecdotally incur considerable out-of-pocket expenses for patients for medical consultations.

This is the third in a series of cross-sectional online Cannabis as Medicine Surveys—conducted in 2016 (CAMS-16) [12], 2018 (CAMS-18) [13] and 2020 (CAMS-20). The first survey was conducted immediately prior to the new legislation and captured a population using illicit cannabis, predominately for pain and mental health conditions, and largely by smoked routes. The 2018–2019 survey identified few participants (2.4%) using prescribed MC, and overall participant demographics, conditions treated and the patterns of use were identical to the 2016 survey, albeit with less smoked and more oral and vaporised routes [13].

We were interested to see whether the increasing uptake of prescribed MC had changed the profile of people using medical cannabis in the community, the range of conditions treated, patterns of use and consumer experiences regarding accessing cannabis within a medical context. It is important for regulators and health care providers to understand community use of medical cannabis, including differences between prescribed and illicit use, which can in turn inform improvements in our regulatory and clinical responses.

The CAMS-20 survey used many of the same questions as previous surveys enabling comparisons over time, but added further questions to better interrogate differences between prescribed and illicit medical cannabis use. As with previous surveys, we use a broad definition of the term ‘medical cannabis’—referring to any legally prescribed or illicit cannabis-based product (including plant matter) used to treat or alleviate the symptoms of a self-identified health condition. The term ‘medicinal cannabis’ specifically refers to a cannabis product prescribed by a medical or nurse practitioner.

## Methods

We used a cross-sectional online survey with a convenience sample of individuals self-reporting cannabis use for therapeutic or medical reasons within the past 12 months. The survey was anonymous, with survey questions (see Additional file 1) examining:

- Demographic characteristics;
- Medical conditions treated with MC;
- Current and lifetime patterns of medical and non-medical cannabis use;
- Self-reported cannabinoid profile of product used (options of ‘mainly THC’, ‘mainly CBD’, THC:CBD combinations, or uncertain).
- Perceived effectiveness (Patient Global Impression of Change (PGIC) [14], a 7-item rating of a patient’s global impression of change in a health condition since commencing medical cannabis treatment from ‘very much worse’ to ‘very much better’); and side effects (symptom checklist) from MC use;
- Consumer perspectives on accessing (or not accessing) prescribed cannabis.

Study data were collected and managed using Research Electronic Data Capture (REDCap), a secure web-based platform [15]. The CAMS-20 survey was ‘live’ online for 5 months (September 2020 to January 2021) and was promoted online using social media and consumer group webpages, at consumer and professional forums, and through a number of private medical cannabis clinics. Eligibility criteria were: (a) informed consent, (b) aged ≥ 18 years, (c) self-identified as using cannabis or a cannabis-based product for a medical purpose within the previous 12 months, and (d) resident in Australia.

Participant responses were grouped and analysed according to their source of cannabis products within the past year:

- ‘Prescribed only’ (PO) group refers to participants who only used prescribed MC,
- ‘Illicit only’ (IO) group refers to participants who only used illicit MC products,
- ‘Prescribed and illicit’ (P + I) group refers to participants who used both prescribed and illicit MC products.

For certain analyses (see below), the PO and P + I groups are collapsed into one group, referred to as the ‘Prescribed’ group or participants.

Only valid responses were analysed, with no imputation of missing data. As the number of valid responses varied across different survey items, frequencies are reported alongside the number of valid responses. Single-level regressions were used to analyse the data, Gaussian for continuous outcomes (e.g. age, cost), binary logistic for two-level categorical outcomes (e.g. relationship status, education), multinomial logistic for greater than two-level outcomes (e.g. main condition treated, side effects), and cumulative link models for ordinal outcomes (e.g. change in tobacco, alcohol, or medication use). The primary predictor variable in these regressions was user type, a three-level categorical predictor indicating whether the respondent was in the PO, IO, or P + I groups within the past year. Estimated marginal means were used to perform group contrasts within each regression model. Two types of comparisons using the three groups were made: (i) a three-way comparison involving all three pairwise combinations of the three groups (PO vs P + I, PO vs IO, P + I vs IO) or (ii) a two-way comparison, comparing ‘Prescribed’ (PO and P + I groups) with the IO group. The Benjamini–Hochberg procedure was used to control the false discovery rate [16]. Statistical analyses were performed in R version 4.0.3 [17] using the tidyverse [18], nnet [19], ordinal [20] and emmeans packages [21].

## Results

### Participants

Of the 2152 respondents who commenced the survey, 188 did not meet eligibility criteria and 40 did not provide consent. Data were excluded for 144 respondents who provided no further information beyond demographics questions, 176 respondents who did not indicate a medical condition for which they use MC and 4 respondents who provided duplicate entries. Data are reported for the remaining 1600 respondents, of whom 1240 (77.5%) completed all survey questions.

Most participants (*n* = 999, 62.4%) reported sourcing illicit only MC products (IO group) in the previous 12 months, and 601 (37.5%) accessed prescribed MC—of whom 388 (24.3%) reported using both prescribed and illicit cannabis for medical reasons (P + I group) and 213 (13.3%) reported using only prescribed products (PO group) in the past 12 months.

Demographics and between-group comparisons (Table 1) indicate PO group participants were significantly older than the P + I and IO participants, were more likely to be female, and were less likely to be employed. The proportion of respondents who currently used tobacco was significantly lower in the PO group than either the P + I group or the IO group.

**Table 1:** Demographic characteristics and other substance use among different groups of respondents

Characteristic	Prescribed only (PO)	Prescribed and illicit (P + I)	Illicit only (IO)	Total	Comparisons estimate (95% CI)^⌠^
Age, M (SD), numeric	50.0 (15.8)^a^	43.5 (12.9)^b^	46.8 (14.4)^c^	46.4 (14.3)	PO – P + I: 6.6 (3.6, 9.5)
					PO – IO: 3.2 (0.6, 5.8)
					P + I – IO: -3.4 (-5.4, -1.3)
*Gender, categorical non-binary*, *n* (%)					
Female	127/213 (60%)^a^	159/388 (41%)^bc^	452/999 (45%)^bc^	738/1600 (46%)	PO – P + I: 2.1 (1.3, 3.6)
					PO – IO: 1.8 (1.1, 2.9)
Male	84/213 (39%)^a^	225/388 (58%)^bc^	533/999 (53%)^bc^	842/1600 (53%)	PO – P + I: 0.5 (0.3, 0.8)
					PO – IO: 0.6 (0.4, 0.9)
Other	2/213 (1%)^abc^	4/388 (1%)^abc^	14/999 (1%)^abc^	20/1600 (1%)	–
In relationship, categorical binary, *n* (%)	135/203 (67%)^abc^	230/361 (64%)^abc^	584/943 (62%)^abc^	949/1507 (63%)	–
Aboriginal and/or Torres Strait Islander, categorical binary, *n* (%)	7/213 (3%)^abc^	19/388 (5%)^abc^	37/999 (4%)^abc^	63/1600 (4%)	–
Attained tertiary qualification^±^, categorical binary, *n* (%)	154/213 (72%)^a^	321/388 (83%)^bc^	781/999 (78%)^abc^	1256/1600 (79%)	PO – P + I: 0.5 (0.3, 0.9)
Employed, categorical binary, *n* (%)	87/213 (31%)^a^	199/388 (51%)^bc^	495/999 (50%)^bc^	781/1600 (49%)	PO – P + I: 0.7 (0.4, 1.0)
Has children, categorical binary, *n* (%)	53/213 (25%)^abc^	127/388 (33%)^abc^	295/999 (30%)^abc^	475/1600 (29.7%)	–
*How respondents heard of survey*^*≠*^*, categorical non-binary*, *n* (%)					
Social media	98/213 (46%)^ab^	194/388 (50%)^ab^	672/999 (67%)^c^	964/1600 (60%)	PO – IO: 0.4 (0.3, 0.6)
					P + I – IO: 0.5 (0.4, 0.7)
Online forum	18/213 (8%)^ac^	79/388 (20%)^b^	111/999 (11%)^ac^	208/1600 (13%)	PO – P + I: 0.4 (0.2, 0.8)
					P + I – IO: 2.0 (1.3, 3.2)
Doctor/health care provider	4/213 (2%)^abc^	7/388 (2%)^abc^	6/999 (1%)^abc^	17/1600 (1%)	-
					PO – P + I: 2.8 (1.6, 4.8)
Medical cannabis provider	68/213 (32%)^a^	56/388 (14%)^b^	41/999 (4%)^c^	165/1600 (10%)	PO – IO: 11.0 (6.1, 19.5)
					P + I – IO: 3.9 (2.2, 7.0)
Other sources	25/213 (12%)^abc^	52/388 (13%)^abc^	169/999 (17%)^abc^	246/1600 (15%)	-
Any tobacco use in previous 28 days, categorical binary, *n* (%)	15/179 (8%)^a^	73/309 (24%)^b^	294/844 (35%)^c^	382/1332 (29%)	PO – P + I: 0.3 (0.2, 0.6)
					PO – IO: 0.2 (0.1, 0.3)
					P + I – IO: 0.6 (0.4, 0.8)
Number of days tobacco use in past 28 days among those with any tobacco use, mean (SD), numeric, (*N* = 1403)	23.5 (9.2)^abc^	22.3 (9.5)^abc^	24.0 (7.9)^abc^	23.6 (8.3)	–
Alcohol use in previous 28 days, *n* (%)	98/179 (54%)^abc^	180/309 (58%)^abc^	541/843 (64%)^abc^	819/1331 (61.5)	–
Number of days alcohol use in past 28 days among those with any alcohol use, mean (SD), (*N* = 1403)	9.4 (8.5)^abc^	9.0 (8.5)^abc^	9.5 (8.6)^abc^	9.4 (8.6)	–

Superscript letters a–c indicate statistically significant between-group differences: Pairs of columns that do not share letters are significantly different from each other (*p* < .05). Missing values excluded from denominator when calculating percentages. Some percentages may not total 100% due to rounding. Letters a–c indicate statistically significant between-group differences. Columns that share letters are NOT significantly different. Median (IQR) reported for count variables only. IQR = Interquartile range. **⌠** Estimates are unstandardised effects size for numeric variables and odds ratios for categorical or ordinal variables. Only significant comparisons reported. **Ŧ** Partnered = currently in relationship, including de facto and married; single = not currently in a relationship, including separated, divorced, widowed. ± Includes both trade/vocational and undergraduate/postgraduate university qualifications. **ǂ** Includes home duties, students, unemployed, retired, and on disability pension. ≠ Social media = Facebook, Twitter, Instagram, Snapchat; Online forum = Reddit, Whirlpool, Bluelight; Other sources = friend, consumer support group, Lambert Initiative website, traditional media (radio, TV, newspaper)

### Health conditions

The main health conditions for which participants used MC are summarised in Table 2 (Additional file 2: Table S1 for details of specific conditions), with pain and mental health conditions being most frequent. Respondents whose MC was prescribed (PO and P + I groups) had significantly greater odds of using cannabis to treat a pain condition than IO participants [OR = 1.7 (1.3, 2.1)], whereas users of illicit MC had greater odds of treating a sleep condition [OR = 0.5 (0.3, 0.8)].

**Table 2:** Main conditions treated with prescribed vs illicit medical cannabis

	Prescribed (*n* = 534)	Illicit only (*n* = 888)	OR (95%CI)
Pain	280 (52%)^a^	352 (40%)^b^	**1.7 (1.3, 2.1)**
Mental health/substance use	141 (26%)^a^	274 (31%)^a^	0.8 (0.6, 1.0)
Neurological	36 (7%)^a^	49 (6%)^a^	1.2 (0.8, 2.0)
Sleep	31 (6%)^a^	101 (11%)^b^	**0.5 (0.3, 0.8)**
Gastrointestinal	11 (2%)^a^	24 (3%)^a^	0.8 (0.3, 1.7)
Cancer	11 (2%)^a^	34 (4%)^a^	0.5 (0.2, 1.1)
Other	24 (5%)^a^	54 (6%)^a^	0.7 (0.4, 1.3)

Columns with different superscript letters were significantly different from one another. *n* = 67 Prescribed and *n* = 111 Illicit Only had missing main condition information. Significant differences in bold

### Cannabis use

Lifetime and recent cannabis use details are shown in Table 3. The PO group had significantly higher odds of never having used cannabis prior to medical use relative to either the P + I group or IO groups, and of their cannabis use being exclusively for medical purposes; and significantly lower odds of non-medical cannabis use upon first use of MC. The PO group commenced regular cannabis use later in life than the P + I and IO groups. Most participants reported using MC on a daily basis, and participants estimated that 88% of their total cannabis use was for medical reasons—significantly higher among the PO group (96%) than the P + I (92%) and IO (85%) groups. Participants reported spending a mean (± SD) of $74 ± 72 (Median = $40, IQR: $7, $100) per week on medical cannabis—significantly higher in the P + I group ($114 ± 77.4; Median = $100, IQR: $50, $155), than the PO ($79.2 ± 61.6; Median = $60, IQR: $35, $100) and IO groups ($58.6 ± 65.4; Median = $40, IQR: $7, $100) (see Table for statistical comparisons).

**Table 3:** Current and lifetime patterns of cannabis use

Characteristic	Prescribed only	Prescribed and illicit	Illicit only	Total	Comparisons estimate (95% CI)^⌠^
*Cannabis use before medical use, categorical non-binary, n *(%)					
Never used cannabis before medical use	126/186 (68%)^a^	63/320 (20%)^bc^	195/895 (22%)^bc^	384/1401 (27%)	PO – P + I: 8.6 (4.5, 16.4)
					PO – IO: 7.5 (4.4, 13.0)
Used ‘recreationally’ but had ≥ 1 year break before medical use	52/186 (28%)^ab^	123/320 (38%)^abc^	382/895 (43%)^bc^	557/1401 (40%)	PO – IO: 0.5 (0.3, 0.9)
					PO – P + I: 0.1 (0.0, 0.2)
Was using recreationally when started using medically	8/186 (4.3%)^a^	134/320 (42%)^bc^	318/895 (36%)^bc^	460/1401 (33%)	PO – IO: 0.1 (0.0, 0.3)
*Age first tried cannabis for any reason, numeric, *(*N* = 1405)					
Mean (SD)	33.2 (21.5)^a^	20.1 (10.7)^bc^	21.7 (13.8)^bc^	22.9 (15.0)	PO – P + I: 13.2 (10.0, 16.3)
Median (IQR)	21 (16, 54)	16 (15, 20)	17 (15, 20)	17 (15, 21)	PO – IO: 11.5 (8.7, 14.3)
*Age first regular cannabis use any reason, numeric, *(N = 1405)					
Mean (SD)	35.4 (23.7)^a^	27.0 (15.1)^bc^	28.4 (17.3)^bc^	29.0 (18.0)	PO – P + I: 8.4 (4.5, 12.4)
Median (IQR)	33 (18, 56)	22 (18, 33)	23 (18, 38)	24 (18, 39)	PO – IO: 11.5 (3.5, 10.4)
*Age first regular cannabis use for medical reason, numeric, *(*N* = 1402)					
Mean (SD)	41.3 (22.6)^a^	36.1 (14.7)^bc^	35.5 (18.2)^bc^	36.4 (18.2)	PO – P + I: 5.2 (1.2, 9.2)
Median (IQR)	46 (28, 59)	35 (26, 45)	35 (23, 49)	35 (24, 50)	PO – IO: 5.8 (2.3, 9.4)
Never used cannabis regularly for any reason, binary categorical, *n* (%)	32/187 (17%)^a^	13/322 (4%)^bc^	63/896 (7%)^bc^	108/1405 (8%)	PO – P + I: 4.9 (2.2, 11.0)
					PO – IO: 2.7 (1.6, 4.7)
*Number of days in previous *28* used cannabis for any reason, numeric, *(*N* = 1403)					
Mean (SD)	21.8 (10.0)^a^	24.9 (6.7)^bc^	20.2 (10.2)^bc^	21.5 (9.7)	PO – P + I: -3.1 (-5.2, -1.0)
Median (IQR)	28 (15, 28)	28 (12, 28)	28 (12, 28)	28 (15, 28)	P + I – IO: 4.7 (3.2, 6.2)
*Number of days in previous *28* used cannabis for medical reasons, numeric, *(*N* = 1402)					
Mean (SD)	21.3 (10.2)^a^	23.8 (8.0)^b^	18.7 (10.8)^c^	20.2 (10.4)	PO – P + I: -2.5 (-4.7, -0.2)
					PO – IO: 2.7 (0.7, 4.6)
Median (IQR)	28 (14, 28)	28 (24, 28)	25 (7, 28)	28 (12, 28)	P + I – IO: 5.1 (3.6, 6.7)
Estimated proportion of cannabis use for medical reasons, numeric, Mean (SD), (*N* = 1392)	96.1 (14.2)^a^	91.9 (14.8)^b^	84.6 (21.4)^c^	87.8 (19.7)	PO – P + I: 4.2 (0.0, 8.5)
					PO – IO: 11.5 (7.8, 15.2)
					P + I – IO: 7.2 (4.2, 10.2)
100% of cannabis use for medical reasons, binary categorical, n (%)	146/186 (79%)^a^	177/322 (55%)^b^	378/884 (43%)^c^	701/1392 (50%)	PO – P + I: 3.0 (1.8, 4.9)
					PO – IO: 4.9 (3.1, 7.6)
					P + I – IO: 1.6 (1.2, 2.2)
*Weekly cost of medical cannabis, numeric, *(*N* = 1395)					
Mean (SD)	$79.2 ($61.6)^a^	$114.0 ($77.4)^b^	$58.6 ($65.4)^c^	$74.0 ($71.6)	PO – P + I: -35.5 (-49.7, -19.6)
					PO – IO: 20.6 (7.4, 33.7)
Median (IQR)	$60 ($35, $100)	$100 ($50, $155)	$40 ($7, $100)	$40 ($7, $100)	P + I – IO: 55.2 (44.6, 65.8)
*Weekly cost of medical cannabis with respondents who did not pay excluded, numeric, *(*N* = 1405)					
Mean (SD)	$81.4 ($61.0)^ac^	$118.0 ($76.0)^ab^	$73.2 ($65.4)^ac^	$85.9 ($70.2)	PO – P + I: -36.5 (-51.7, -21.3)
Median (IQR)	$65 ($40, $100)	$100 ($50, $170)	$100 ($50, $155)	$50 ($25, $100)	P + I – IO: 44.7 (33.7, 55.7)

Superscript letters a–c indicate statistically significant between-group differences: Pairs of columns that do not share letters are significantly different from each other (*p* < 0.05). Missing values excluded from denominator when calculating percentages. Some percentages may not total 100% due to rounding. Letters a–c indicate statistically significant between-group differences. Columns that share letters are NOT significantly different. Median (IQR) reported for count variables only. IQR = Interquartile range. **⌠** For each outcome, pairwise comparisons were made for all three groups: PO – P + I, PO-IO, and P + I – IO. Estimates are unstandardised effects size for numeric variables and odds ratios for categorical or ordinal variables. Only significant comparisons reported

Within the P + I group (*n* = 388), only 34.5% (134) had used illicit supplies in the preceding 2 weeks and reported intending to continue regularly using illicit supplies, while 26% (101) indicated that they had used illicit supplies occasionally and would use as required, and 39.4% (153) indicated that they had not recently used illicit MC and did not plan on resuming illicit products. Among P + I group, the main reasons for continuing to use illicit supplies were: cost of prescribed MC (79.3%, 73/92), to ensure an adequate supply (51.1%, 47/92), to improve effectiveness (48.9%, 45/92) and the cost of medical consultation fees (38%, 35/92).

**Fig. 1:**
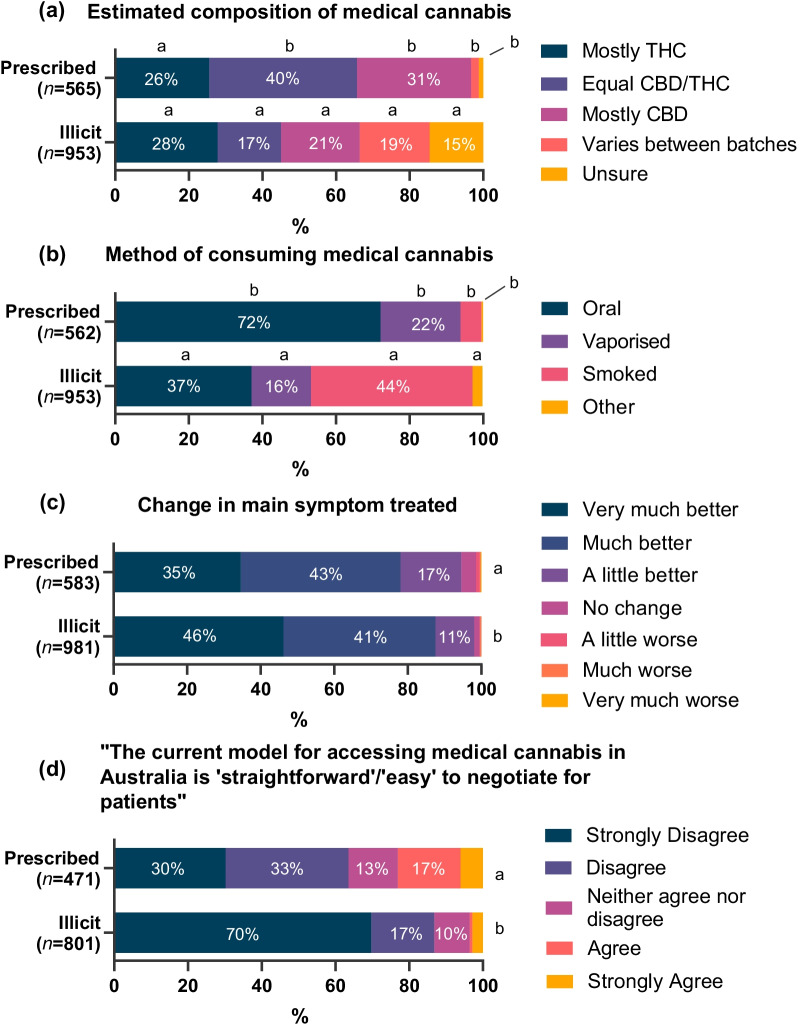
**a** Estimated composition of medical cannabis; **b** main method of administering; **c** change in main condition following treatment with medical cannabis (PGIC); **d** consumer perception of ease of access to medical cannabis treatment. Cells without percentages were all ≤ 6%. In panels **a** and **b**, outcomes were treated as unordered categorical. In these panels, portions of graph of the same shade but with different letters are significantly different proportions. In panels **c** and **d**, outcome was treated as ordinal and different letters indicate significantly different odds of indicating a higher category vs a lower category

Respondent’s estimates of the composition of their MC are shown in Fig. 1(a). Few participants (3%, 19/565) accessing prescribed products reported uncertainty regarding the composition of their MC (i.e. unsure of the composition or that it varied between batches) compared to ‘illicit only’ participants (34%, 343/953) (OR = 0.1; CI: 0.0, 0.1; *p* < 0.001).

The main route of administration is reported in Fig. 1(b). Prescribed participants were more likely than IO participants to use oral routes [72% (405/562) vs 37% (355/953), respectively; OR = 4.3; CI: 3.3, 5.8; *p* < 0.001] and vaporised routes [22% (122/562) vs 16% (154/953); OR = 1.4; CI: 1.0, 2.0; *p* = 0.04], whereas IO participants were more likely to smoke their medical cannabis by joints or ‘bongs’ [44% (418/953) vs 6% (32/562); OR = 12.9; CI: 8.1, 20.7; *p* < 0.001].

Maintaining regular access to MC was a problem for many participants, with 624/1520 (42%) indicating they were unable to access MC at some point during the past month, more often reported by IO participants [440/953 (46.2%)], than those using prescribed cannabis [184/567 (32.4%); OR = 1.79; CI: 1.1, 2.9; *p* = 0.03].

### Safety and effectiveness

The overwhelming majority of participants (95% Prescribed, 98% Illicit) reported that their main health condition had improved using the Patient Global Impression of Change since commencing MC (Fig. 1c). The odds of respondents giving a higher rating vs a lower rating for the amount of improvement in their condition were greater for illicit than prescribed cannabis (OR = 1.7, CI: 1.4, 2.1, *p* < 0.001).

Side effects were most frequently reported as ‘mild and tolerable’, with the five most common side effects being dry mouth [742/1308 (56.7%)], increased appetite [653/1309 (49.9%)], drowsiness [640/1308 (48.9%)], fatigue [344/1308 (26.3%)] and eye irritation [334/1308 (26%)] (Additional file 2: Table S2a). IO participants had significantly greater odds of reporting more severe nasal (OR = 1.9; 95%CI 1.1, 3.4, *p* = 0.03) and respiratory (OR = 2.1; 95%CI 1.5, 3.1, *p* < 0.001) side effects, while prescribed participants reported significantly more severe dizziness (OR = 0.7; 95%CI 0.5, 0.9, *p* = 0.008) and nausea (OR = 0.5; 95%CI 0.3, 0.8, *p* = 0.004) (Additional file 2: Table S2b).

### Involvement of health providers

Among participants prescribed MC, 25.3% were prescribed by a general practitioner (152/600), 10.3% by a medical specialist (*n* = 62) and 63.8% by a private ‘medical cannabis clinic’ (*n* = 383). Prescribed participants reported having used prescribed cannabis for 8.8 ± 8.5 months (median 6, IQR = 3–12), with a median interval of 3 weeks (IQR = 2–4) from initial medical consultation to accessing their first dose.

The reasons cited by IO participants for not accessing prescribed cannabis were: too expensive (46.9%, 468/999); not knowing any medical practitioners willing to prescribe (35.9%, *n* = 358), their own medical practitioner being unwilling to prescribe (25.3%, *n* = 252), unaware that cannabis could be prescribed (12.1%, *n* = 121), concerns with confidentiality (18.5%, *n* = 184), and 15.9% (*n* = 159) indicated that they preferred illicit over prescribed products.

The overwhelming majority (92.6%, 452/488) of prescribed participants had discussed their MC use with some or most of their health care providers, significantly greater than IO participants (35.5%, 546/847) (OR = 5.0, 95%CI 4.0, 6.3, *p* < 0.001). Respondents discussed their MC use most frequently with their general practitioner (89.5%, 891/996), medical specialist (59.4%, 592/996), counsellor/psychologist (37.4%, 373/996) and pharmacist (25.4%, 253/996).

Few participants indicated that the current model for accessing MC was ‘straightforward or easy’ to negotiate (Fig. 1d), with only 3% of IO participants agreeing or strongly agreeing with the statement, and 24% of those accessing prescribed cannabis (OR = 0.2; CI: 0.2, 0.3; *p* < 0.001).

### Cannabis use during COVID

Most respondents [1093/1399 (78%)] reported no change in their cannabis use since the COVID-19 pandemic, although 15.0% (210/1399) reported increased MC use and 6.9% (96/1399) increased non-medical use, with no significant group differences.

## Discussion

Our findings indicate a substantial increase in the use of prescribed MC products in Australia in 2020 compared to our earlier surveys, consistent with recent regulatory data [11]. Use of a prescribed product was reported by 37.5% of respondents, a dramatic increase relative to our CAMS in 2016 [13] (< 1%) and 2018 [9] (< 3%).

This is the first opportunity to examine differences between prescribed and illicit medical cannabis use in Australia, with a range of differences between people using prescribed versus illicit MC products. Individuals only using prescribed cannabis in the previous year (PO group) were older, more likely to be female, less likely to be employed, with less lifetime non-medical cannabis use and later initiation of cannabis use (for any reason). In contrast, the characteristics of P + I and IO groups were quite similar, suggesting a recent transition in some illicit users towards prescribed MC. Of note, most P + I group participants did not intend to continue regular illicit cannabis use.

Our findings suggest some harm reduction advantages in prescribed over illicit cannabis use including safer routes of administration, greater certainty of access and known THC/CBD composition, and better communication with health care providers. These benefits suggest further efforts are warranted to transition patients from illicit to regulated, quality-controlled, cannabis products.

The reasons cited for not using prescribed products included difficulties in finding a prescriber, perceived unaffordability of prescribed products, and, to a lesser degree, concerns regarding confidentiality or stigma. Only a small proportion (15.9%) of those only using illicit medical cannabis indicated a preference for illicit cannabis products. Access to medical practitioners skilled and willing to prescribe MC clearly remains a problem for many Australians, consistent with findings of a recent Senate Inquiry [22] despite the increasing training opportunities for clinicians. Further research examining medical practitioner perspectives is required to better understand and address these barriers.

Most consumers—even those currently accessing prescribed cannabis—find the existing access pathways difficult to navigate, suggesting the need for better consumer resources, and for further regulatory reforms. Cost remains a barrier for many consumers, with the unlicensed nature of current products preventing their subsidy under the Pharmaceutical Benefits Scheme, the framework for subsidised medications in Australia.

The study has inherent limitations. Reliance on self-report data may lead to inaccurate information around diagnostic conditions, effectiveness and adverse events. Difficulties in reliably reporting doses and uncertainties in THC and CBD composition of illicit cannabis products prevent further examination of these factors. Online surveys employing convenience sampling are also likely to encounter selection bias towards recruiting people with favourable experiences of MC. Nevertheless, comparison of demographic data for the prescribed participants in our sample is comparable (mean age = 45.8 years; male gender 51.4%) with regulatory data of medicinal cannabis approvals [9] (median age group reported 45–52, male gender in 46.3% of approvals in 2020). Furthermore, our design does not allow us to confidently estimate the proportion of people using prescribed versus illicit MC products, nor the number of people using MC in Australia.

## Conclusions

The introduction of a regulatory framework for medicinal cannabis in late 2016 has resulted in a considerable uptake of prescribed MC use by 2020 after several years of limited access. The regulatory framework appears to have attracted consumers with little or no prior illicit cannabis use (for medical or non-medical reasons), as well as transitioning a group of patients from illicit to prescribed MC products. The benefits of prescribed MC include greater certainty regarding dose and cannabinoid composition, more reliable access to supplies, safer routes of administration and greater communication with health care professionals (important given the potential for drug–drug interactions with cannabis products). Nevertheless, some consumers clearly continue to find the regulatory framework difficult to navigate, and the cost of prescribed products to the consumer remains a barrier to many, suggesting that further refinements to the Australian treatment framework may be beneficial. Further research and strategies are required to address the barriers consumers report in accessing medical practitioners willing to prescribe medicinal cannabis.

## Supplementary Information

**Additional file 1**. Consumer perspectives regarding medical cannabis. (a) Estimated composition of medical cannabis mainly used; (b) main method of administering medical cannabis; (c) change in main condition following treatment with medical cannabis (PGIC); (d) consumer perception of ease of access to medical cannabis treatment. The legend for Figure 1 should read as follows: Legend: Cells without percentages were all ≤6%. In panels (a) and (b), outcomes were treated as unordered categorical. In these panels portions of graph of the same shade but with different letters are significantly different proportions. In panels (c) and (d) outcome was treated as ordinal and different letters indicate significantly different odds of indicating a higher category vs a lower category.

**Additional file 2: Table S1.** Main conditions treated with prescribed and illicit cannabis based on user type. **Table S2a.** Rates of side effects. **Table S2b.** Results of ordinal logistic regressions testing differences between prescribed and illicit only users in odds of endorsing more severe side effects.

## Data Availability

The survey questions are available as an online supplement. The data sets used and/or analysed during the current study are available from the corresponding author on request.
