# Supplementary material for: Medical cannabis use in Australia: consumer experiences from the online cannabis as medicine survey 2020 (CAMS-20)
Source: Harm Reduct J. 2022 Jul 30;19:88. doi: 10.1186/s12954-022-00666-w (PMC9338505; doi:10.1186/s12954-022-00666-w)
Supplement: Supplementary file 1 — Additional file 1. Consumer perspectives regarding medical cannabis. (a) Estimated composition of medical cannabis mainly used; (b) main method of administering medical cannabis; (c) change in main condition following treatment with medical cannabis (PGIC); (d) consumer perception of ease of access to medical cannabis treatment. The legend for Figure 1 should read as follows: Legend: Cells without percentages were all ≤6%. In panels (a) and (b), outcomes were treated as unordered categorical. In these panels portions of graph of the same shade but with different letters are significantly different proportions. In panels (c) and (d) outcome was treated as ordinal and different letters indicate significantly different odds of indicating a higher category vs a lower category. [file 12954_2022_666_MOESM1_ESM.pdf]

# Cannabis as Medicine Survey: 2020

---

As researchers from the University of Sydney we are launching an Australia-wide online survey of people who have used cannabis for MEDICAL (therapeutic) purposes within the last 12 months. Similar surveys were conducted in 2016 and 2018, and we aim to see how things have changed since the last survey.

The survey will be anonymous and confidential. You have been allocated a user ID code that in no way allows for your responses to be matched to personal and/or identifying data.

The survey asks about:

1. You: your age, gender, level of education and current living and work situations

2. Conditions you have used medicinal cannabis to treat/cure in the last 12 months

3. How you access and administer cannabis for medical purposes: LEGAL (PRESCRIBED) OR ILLICIT (NON-PRESCRIBED). These categories are in line with Federal legislation.

4. The extent to which you experience benefits (e.g. symptom relief) and/or problems (e.g. side effects, social or legal problems) from your use of medicinal cannabis.

5. How you would like cannabis products to be available for medical reasons in the future

6. Your general health and recent use of other substances.

PLEASE NOTE: CBD-only products (e.g. CBD oils or capsules) fall within the meaning of "medicinal cannabis.

For further details and instructions please read the participant information statement attached here.

[Attachment: "ParticipantInformationStatement_CAMS20_v2.pdf"]

---

| | |
|---|---|
| Please indicate your eligibility for the study below: | ○ I am an Australian resident over the age of 18 who has used cannabis for MEDICAL reasons in the last 12 MONTHS. I understand that "medicinal cannabis" includes CBD-only products. Click here to confirm. |
| Informed consent | ○ I have read the Participant Information Statement and understand the nature of my involvement in this study. I understand that I can withdraw from involvement at any time by exiting the server browser. In this case my data will not be saved or analysed. I also understand that I can save my responses and return to complete this study at a later time. To do so I must record the User ID code provided when I select the "save and return" option at the end of any page. Click here to agree. |

**SECTION 1**

**ABOUT YOU**

**Please provide some basic information about yourself.**

Gender
- ◯ Male
- ◯ Female
- ◯ Other

Please tell us which gender identity you most readily identify as.

__________________________ (please specify)

Age

__________________________ (Years old)

Which state/territory do you live in?
- ◯ New South Wales
- ◯ Victoria
- ◯ Queensland
- ◯ Northern Territory
- ◯ Western Australia
- ◯ South Australia
- ◯ Tasmania
- ◯ Australian Capital Territory

What is the post code of your current address?

__________________________

Are you of Aboriginal or Torres Strait Islander origin?
- ◯ No
- ◯ Yes, Aboriginal
- ◯ Yes, Torres Strait Islander
- ◯ Yes, both Aboriginal and Torres Strait Islander

What is the highest level of education you have completed?
- ◯ Primary School
- ◯ Secondary/High school
- ◯ Trade or Vocational training (e.g. TAFE or college)
- ◯ Undergraduate University degree (e.g. Bachelors, Honours)
- ◯ Postgraduate University degree (e.g. Masters, PhD)
- ◯ Other

How else would you describe your highest level of education?

__________________________ (please describe)

Which option best describes your current employment status?
- ◯ Full time work
- ◯ Part time work
- ◯ Home duties
- ◯ Unemployed
- ◯ Retired
- ◯ Disability pension
- ◯ Student
- ◯ Other

How would you best describe your current employment status?

__________________________

| | |
|---|---|
| Did you employment status change as a result of the COVID-19 pandemic? | ◯ Yes<br>◯ No |
| Which option best describes your employment status prior to the COVID-19 pandemic? | ◯ Full time work<br>◯ Part time work<br>◯ Home duties<br>◯ Unemployed<br>◯ Retired<br>◯ Disability pension<br>◯ Student<br>◯ Other |
| How would you best describe your current employment status? | __________________________________ |
| Which of the following best describes the sector you have been employed in for the majority of your working life? | ◯ Hospitality<br>◯ Retail<br>◯ Office<br>◯ Healthcare<br>◯ Construction<br>◯ Education<br>◯ Manufacturing<br>◯ Transport<br>◯ Mining<br>◯ Law enforcement<br>◯ Never employed<br>◯ Other |
| How would you best describe the sector you have been employed in for the majority of your working life? | __________________________________ |
| On how many days of the last 28 days did you engage in PAID work (do not include voluntary work) and/or study (school, TAFE, university or vocational training)? | __________________________________<br>(Please enter a value from 0-28 (Full time = 20 days)) |
| What is your current relationship status? | ◯ Single (not currently in a relationship)<br>◯ Partnered (currently in a relationship)<br>◯ Would rather not say |
| In the last 28 days, have you been the primary care giver for, or living with, any child/children under the age of 18? | ◯ Yes<br>◯ No |
| Where did you first hear about this Survey? | ◯ Facebook<br>◯ Other social media (e.g. Instagram, Twitter, Snapchat)<br>◯ Online forum (e.g. Reddit, Whirlpool, Bluelight)<br>◯ A friend<br>◯ Medicinal cannabis provider<br>◯ Consumer support group<br>◯ Lambert Initiative website<br>◯ Doctor/healthcare provider<br>◯ Private cannabis clinic (e.g. CA clinic, Tetra health, etc.)<br>◯ Media (newspaper, radio, TV)<br>◯ Other<br>(select one option) |

From what other source did you first hear about this survey?

__________________________________
(please describe)

**SECTION 2**

**ACCESS TO MEDICINAL CANNABIS**

**The following questions ask you about the way you access your medicinal cannabis - through legal prescribed routes or through other routes. Your responses are anonymous and confidential.**

| | |
|---|---|
| Have you accessed legal medicinal cannabis prescribed by a doctor in Australia? Prescribed medicinal cannabis products are obtained by filling a script at a pharmacy or a chemist. | ◯ Yes<br>◯ No |
| You have indicated that you have not accessed legal medicinal cannabis prescribed by a doctor in Australia.<br><br>Please describe the main reasons for you not accessing medicinal cannabis from a medical practitioner | ☐ I did not know I could legally access medicinal cannabis from a doctor<br>☐ I do not know a medical practitioner who is willing to prescribe medicinal cannabis<br>☐ My medical practitioner is not interested or is unwilling to prescribe<br>☐ Medicinal cannabis supplies are too expensive for me to afford<br>☐ I prefer using illicit cannabis<br>☐ I prefer to keep my medicinal cannabis use confidential from healthcare providers<br>☐ Other reason<br>(You can select more than one option.) |
| Please describe the other reason you don't access legal (prescribed) medicinal cannabis | ________________________________ |
| Please indicate whether you are a current or a previous legal (prescribed) medicinal cannabis user. Please see the definitions below for current and previous use.<br><br>Current use: I have used prescribed medicinal cannabis in the last 2 weeks, and plan to continue using in the future.<br><br>Previous use: I have used prescribed medicinal cannabis in the last 12 months, but I stopped using more than 2 weeks ago, and do not plan to continue using.<br><br>Occasional use: I have used prescribed medicinal cannabis in the last 12 months, as and when required, and plan to continue using in the future. | ◯ I am currently using legal medicinal cannabis<br>◯ I have previously used legal medicinal cannabis<br>◯ I occasionally use legal medicinal cannabis |

| | |
|---|---|
| Please describe the reasons you discontinued your use of legal (prescribed) medicinal cannabis. | ☐ Medicinal cannabis wasn't improving my symptoms<br>☐ Medicinal cannabis supplies became too expensive for me to afford<br>☐ Medicinal cannabis became difficult to access<br>☐ I became concerned about mobile drug-testing<br>☐ I became concerned about side-effects<br>☐ My medicinal practitioner became uninterested or unwilling to prescribe<br>☐ I preferred using illicit cannabis<br>☐ I preferred to keep my medicinal cannabis use confidential from healthcare providers<br>☐ Other<br>(You can select more than one option.) |
| Why else did you discontinue using legal (prescribed) medicinal cannabis? | __________________________________________ |
| Do you have experience accessing medicinal cannabis through illicit (non-prescribed) avenues?<br><br>These are avenues which do not involve meeting with a GP or specialist, who gives a script for medicinal cannabis. | ◯ Yes; I have used both prescribed and non-prescribed products<br>◯ No; I have used only prescribed products |
| Please indicate whether you are a current or a previous ILLICIT (non-prescribed) medicinal cannabis user. Please see the definitions below for current and previous use.<br><br>Current use: I have used illicit (non-prescribed) medicinal cannabis in the last 2 weeks, and plan to continue using in the future.<br><br>Previous use: I have used illicit (non-prescribed) medicinal cannabis in the last 12 months, but I stopped using more than 2 weeks ago, and do not plan to continue using.<br><br>Occasional use: I have used prescribed medicinal cannabis in the last 12 months, as and when required, and plan to continue using in the future. | ◯ I am currently using illicit (non-prescribed) medicinal cannabis<br>◯ I have previously used illicit (non-prescribed) medicinal cannabis<br>◯ I occasionally use illicit (non-prescribed) medicinal cannabis |
| Please describe the reasons you discontinued your use of illicit (non-prescribed) medicinal cannabis. | ☐ Medicinal cannabis wasn't improving my symptoms<br>☐ Medicinal cannabis supplies became too expensive for me to afford<br>☐ Medicinal cannabis became difficult to access<br>☐ I became concerned about mobile drug-testing<br>☐ I became concerned about side-effects<br>☐ I preferred using prescribed cannabis<br>☐ Other<br>(You can select more than one option.) |
| Why else did you discontinue using illicit (non-prescribed) medicinal cannabis? | __________________________________________ |

| | |
|---|---|
| Why do you use illicit (non-prescribed) MC products in addition to a legal (prescribed) MC product? | ☐ To ensure adequate supply<br>☐ Cost of legal products<br>☐ Difficulty obtaining a prescription<br>☐ Cost of consultation fees<br>☐ To improve effectiveness<br>☐ ACT only: I'm able to legally grow my own<br>☐ Other<br>(You can select more than one option.) |
| Please describe which other reason describes your reason for using both illicit (non-prescribed) and legal (prescribed) medicinal cannabis products | __________________________________ |
| For each of the following please indicate whether you prefer illicit (non-prescribed) or legal (prescribed) medicinal cannabis | |
| Consistency of dose from batch to batch | ◯ Strongly prefer illicit<br>◯ Prefer illicit<br>◯ No preference<br>◯ Prefer prescribed<br>◯ Strongly prefer prescribed<br>(select one option) |
| Ease of access (getting supplies) | ◯ Strongly prefer illicit<br>◯ Prefer illicit<br>◯ No preference<br>◯ Prefer prescribed<br>◯ Strongly prefer prescribed<br>(select one option) |
| Cost | ◯ Strongly prefer illicit<br>◯ Prefer illicit<br>◯ No preference<br>◯ Prefer prescribed<br>◯ Strongly prefer prescribed<br>(select one option) |
| Effectiveness (how well it treats my health condition) | ◯ Strongly prefer illicit<br>◯ Prefer illicit<br>◯ No preference<br>◯ Prefer prescribed<br>◯ Strongly prefer prescribed<br>(select one option) |
| Side effects | ◯ Strongly prefer illicit<br>◯ Prefer illicit<br>◯ No preference<br>◯ Prefer prescribed<br>◯ Strongly prefer prescribed<br>(select one option) |
| Security (avoiding the possibility of prosecution/charge) | ◯ Strongly prefer illicit<br>◯ Prefer illicit<br>◯ No preference<br>◯ Prefer prescribed<br>◯ Strongly prefer prescribed<br>(select one option) |

| | |
|---|---|
| If you could access legal medicinal cannabis, which form of administration would you prefer to use? | ◯ Oral (tablet or capsule)<br>◯ Oral edibles (cake, cookie etc.)<br>◯ Oral liquid concentrate (oil, tincture, etc.)<br>◯ Oral liquid as a fresh juice<br>◯ Oral mouth spray<br>◯ Nasal application (through nose)<br>◯ Smoked as a 'joint' (cigarette)<br>◯ Smoked using a 'dry' pipe (plastic, metal, glass)<br>◯ Smoked using a water pipe or 'Bong'<br>◯ Smoked by 'dabbing' or 'spotting'<br>◯ Inhaled using a vaporiser<br>◯ Suppository<br>◯ Topical (cream or patch applied to the skin)<br>◯ Other<br>(select one option) |
| What other form of administration would be your preferred choice for using medicinal cannabis? | __________________________________ |
| How would you prefer to legally access medicinal cannabis products?<br><br>Note: We recognise that "I grow my own" is a legal practice in the ACT. | ◯ Grow your own<br>◯ Trade/buy from a friend<br>◯ From a special licensed cannabis dispensary<br>◯ From a pharmacy like any other medication<br>◯ No preference, so long as a regular supply is accessible<br>◯ Don't know |
| Please provide an estimation of how much you would be willing to pay per WEEK for legal access to medicinal cannabis?  Please round up to the nearest dollar, and use a whole number only. | __________________________________<br>(In Australian dollars) |

**SECTION 3**

**REASONS FOR USING MEDICINAL CANNABIS**

**The following questions ask about:**

**a) The medical condition(s) for which you use PRESCRIBED and/or ILLICIT medicinal cannabis AND (b) the specific symptoms for which you use PRESCRIBED and/or ILLICIT medicinal cannabis.**

**Please keep in mind that a medical condition refers to an injury, illness, or disease that affects your physical and/or mental health. In contrast, symptoms are what people experience (e.g. nausea, pain) and are often not specific to a medical condition.**

**EXAMPLE: Some people may have cancer as their main health CONDITION for using medicinal cannabis, and use cannabis for the relief of SYMPTOMS of pain, poor appetite, and anxiety.**

Why did you start using medicinal cannabis? Please select all options that apply.

- ☐ My usual medications were not working on my long-term or chronic illness
- ☐ I preferred a natural alternative to my usual medication
- ☐ Side effects of my usual medications
- ☐ While using recreationally I discovered cannabis had medicinal benefits
- ☐ It was recommended to me by a friend or family member
- ☐ It was recommended to me by a health professional (GP, specialist, allied health, holistic etc)
- ☐ Willingness to explore alternative health management strategies
- ☐ Other

Please describe which other reason(s) were behind your use of medicinal cannabis

______________________________________

**The following questions refer to your use of LEGAL (PRESCRIBED) medicinal cannabis, obtained on script from a pharmacy.**

You have indicated that you have accessed legal medicinal cannabis prescribed by a doctor in Australia.

Who is the MAIN prescriber of your medicinal cannabis?

- ◯ GP
- ◯ Medical specialist
- ◯ Private medicinal cannabis clinic
- ◯ Other

(select one option)

---

What is the name of the "other" medicinal cannabis prescriber you have used to access your medicinal cannabis?

__________________________________

---

What is the name of the MAIN private clinic you use to access your prescribed medicinal cannabis?

- ◯ Cannabis Access Clinics (CA Clinics)
- ◯ Medical Cannabis Services (MCS Clinics)
- ◯ Cannabis Doctors Australia (CDA Clinics)
- ◯ Tetra Health Clinics
- ◯ Other

---

Which other private medicinal cannabis clinic not listed above is the MAIN way you access your legal medicinal cannabis?

__________________________________

---

For how long have/had you been accessing legal (prescribed) medicinal cannabis?

Please answer in number of MONTHS

____________________________
(in months (please use numbers). If less than 1 month, please indicate e.g. 2 weeks = 0.5, 3 weeks = 0.75)

---

Please estimate how long it took you to get your first dose of medicinal cannabis from the time you approached your medicinal cannabis doctor.

Please answer in number of WEEKS

If it took more than six months please enter '25'

____________________________
(time in weeks (please use numbers))

---

Have you used PRESCRIBED medicinal cannabis to treat a condition from any of the following groups?

You may select more than one option

- ☐ Cancer
- ☐ Gastro-intestinal
- ☐ Mental health and/or substance use
- ☐ Neurological
- ☐ Pain
- ☐ Sleep
- ☐ Other

(select all that apply)

---

Please indicate which of the following cancer conditions you have used PRESCRIBED medicinal cannabis to treat.

- ☐ Blood cancers (leukaemia, lymphoma, myeloma)
- ☐ Brain cancers (glioblastoma, neuroblastoma, mesothelioma)
- ☐ Breast cancer
- ☐ Gastrointestinal cancer (bowel, colon, stomach, pancreatic)
- ☐ Lung cancer
- ☐ Reproductive cancers (prostate, cervical, uterine, testicular, ovarian)
- ☐ Skin cancers (melanoma)
- ☐ Other forms of cancer

(select all that apply (limit of three))

| | |
|---|---|
| What other type of cancer have you used PRESCRIBED medicinal cannabis to treat? | __________________________________ |
| Please indicate which of the following gastro-intestinal conditions you have used PRESCRIBED medicinal cannabis to treat. | ☐ Crohn's disease<br>☐ Irritable bowel syndrome<br>☐ Ulcerative collitis<br>☐ Other gastro-intestinal condition<br>(select all that apply (limit of three)) |
| What other type of gastro-intestinal conditions have you used PRESCRIBED medicinal cannabis to treat? | __________________________________ |
| Please indicate which of the following mental health conditions you have used PRESCRIBED medicinal cannabis to treat. | ☐ Addiction (Cannabis)<br>☐ Addiction (Other drugs: including alcohol, opioid, amphetamine)<br>☐ Anxiety Disorder (e.g. generalised anxiety, panic disorder, OCD)<br>☐ Attention Deficit disorder (ADHD)<br>☐ Depression<br>☐ Eating disorders (e.g. anorexia / bulimia/ obesity)<br>☐ Manic Disorder (Bipolar affective disorder)<br>☐ Post-Traumatic Stress Disorder<br>☐ Schizophrenia or other psychosis<br>☐ Other mental health condition<br>(select all that apply (limit of three)) |
| What other type of mental health condition do you use prescribed medicinal cannabis to treat? | __________________________________ |
| Please indicate which of the following neurological conditions you use PRESCRIBED medicinal cannabis to treat.<br><br>Please note: for the purposes of this survey fibromyalgia, neuropathy, and mental illnesses (e.g. depression, anxiety, ADHD, Schizophrenia) are NOT considered to be neuorological disorders. If you clicked on this category in order to indicate one of these conditions you will find fibromyalgia and neuropathy under pain conditions and depression, anxiety, ADHD, and Schizophrenia under mental health and/or substance use conditions. | ☐ Amyotrophic Lateral Sclerosis<br>☐ Autism<br>☐ Dementia (including Alzheimer's)<br>☐ Epilepsy/ Seizure disorder<br>☐ Glaucoma<br>☐ Huntington's disease<br>☐ Multiple Sclerosis<br>☐ Parkinson's Disease<br>☐ Tourette's Syndrome<br>☐ Other neurological condition<br>(select all that apply (limit of three)) |
| What other type of neurological conditions have you used PRESCRIBED medicinal cannabis to treat? | __________________________________<br>(please describe) |

| | |
|---|---|
| Please indicate which of the following pain conditions you have used PRESCRIBED medicinal cannabis to treat | ☐ Arthritis (including rheumatoid or osteoarthritis<br>☐ Back pain<br>☐ Cancer-related pain<br>☐ Fibromyalgia<br>☐ Gynaecological pain<br>☐ Headaches (including migraines)<br>☐ Menstrual pain<br>☐ Neck pain<br>☐ Neuropathy (nerve pain)<br>☐ Spinal cord injury<br>☐ Other type of chronic, non-cancer pain not described above<br>(select all that apply (limit of three)) |
| What other type of pain disorder have you used PRESCRIBED medicinal cannabis to treat? | ____________________________________<br>(please describe) |
| Please indicate which of the following sleep conditions you have used PRESCRIBED medicinal cannabis to treat | ☐ Circadian rhythm sleep disorder (e.g. shift work type)<br>☐ Insomnia (any type)<br>☐ Narcolepsy or other hypersomnia<br>☐ Parasomnias (e.g. sleep walking, nightmares)<br>☐ Sleep apnoea or other sleep-related breathing disorder<br>☐ Sleep-related movement disorder (e.g. restless leg syndrome)<br>☐ Other sleep disorder<br>(select all that apply (limit of three)) |
| What other type of sleep disorder do you use PRESCRIBED medicinal cannabis to treat? | ____________________________________<br>(please describe) |
| Please indicate which of the following other conditions you use PRESCRIBED medicinal cannabis to treat | ☐ Auto-Immune condition (e.g. SLE, Chronic Fatigue disorder)<br>☐ Cardiovascular condition (e.g. poor circulation, ischemic heart disease)<br>☐ Diabetes Mellitus<br>☐ Gynaecological condition (e.g. endometriosis, PMS)<br>☐ AIDS/HIV<br>☐ Infectious disease (e.g. viral hepatitis)<br>☐ Respiratory disease (e.g. asthma, cystic fibrosis)<br>☐ Skin condition (e.g. eczema, psoriasis, dermatitis)<br>☐ Other condition<br>(select all that apply (limit of five)) |
| What other condition have you used PRESCRIBED medicinal cannabis to treat? | ____________________________________<br>(please describe) |

**The following questions ask about the MAIN health condition for which you have used PRESCRIBED cannabis for medical purposes, and how this condition changed. The main health condition I use prescribed medicinal cannabis for is:**

| | MAIN condition |
| --- | --- |
| Addiction (Cannabis) | ○ |
| Addiction (Other drugs: including alcohol, opioid, amphetamine) | ○ |
| Amyotrophic Lateral Sclerosis | ○ |
| Anxiety Disorder (e.g. generalised anxiety, panic disorder, OCD) | ○ |
| Arthritis (including rheumatoid or osteoarthritis | ○ |
| Attention Deficit disorder (ADHD) | ○ |
| Autism | ○ |
| Auto-Immune condition (e.g. SLE, chronic fatigue disorder) | ○ |
| Back pain | ○ |
| Blood cancers (leukaemia, lymphoma) | ○ |
| Brain cancers (glioblastoma, neuroblastoma, mesothelioma) | ○ |
| Breast cancer | ○ |
| Cancer-related pain | ○ |
| Cardiovascular condition (e.g. poor circulation, ischemic heart disease) | ○ |
| Circadian rhythm sleep disorder (e.g. shift work type) | ○ |
| Crohn's disease | ○ |
| Dementia (including Alzheimer's) | ○ |
| Depression | ○ |
| Diabetes Mellitus | ○ |
| Eating disorders (e.g. anorexia / bulimia/ obesity) | ○ |
| Epilepsy/ Seizure disorder | ○ |
| Fibromyalgia | ○ |
| Gastrointestinal cancer (bowel, colon, stomach) | ○ |
| Glaucoma | ○ |

Gynaecological condition (e.g. endometriosis, PMT) ◯

Headaches (including migraines) ◯

Huntington's disease ◯

Infectious disease (e.g. AIDS/HIV, viral hepatitis) ◯

Insomnia (any type) ◯

Irritable bowel syndrome ◯

Lung cancer ◯

Manic Disorder (Bipolar affective disorder) ◯

Menstrual pain ◯

Multiple Sclerosis ◯

Narcolepsy or other hypersomnia ◯

Neck pain ◯

Neuropathy (nerve pain) ◯

[condition_cancer_other] ◯

[condition_gastro_other] ◯

[condition_mentalh_other] ◯

[condition_neuro_other] ◯

[condition_sleep_other] ◯

[condition_pain_other] ◯

Parasomnias (e.g. sleep walking, nightmares) ◯

Post-Traumatic Stress Disorder ◯

Reproductive cancers (cervical, uterine, testicular, ovarian) ◯

Respiratory disease (e.g. asthma, cystic fibrosis) ◯

Schizophrenia or other psychosis ◯

Skin cancers (melanoma) ◯

Skin condition (e.g. eczema, psoriasis, dermatitis) ◯

Sleep apnoea or other sleep-related breathing disorder ◯

Sleep-related movement disorder (e.g. restless leg syndrome) ◯

Spinal cord injury ◯

Tourette's Syndrome ◯

Ulcerative collitis ◯

[condition_other_other] ◯

Please check the option that best describes how your MAIN health condition for which you are PRESCRIBED medicinal cannabis is now, compared with how it was before using medicinal cannabis.

◯ Very much better
◯ Much better
◯ A little better
◯ No change
◯ A little worse
◯ Much worse
◯ Very much worse
(select one option)

**Please indicate which of the following SYMPTOMS you have used PRESCRIBED medicinal cannabis to manage in the past 12 months. Please keep in mind that a medical condition refers to an injury, illness, or disease that affects your physical and/or mental health. In contrast, symptoms are what people experience (e.g. nausea, pain) and are often not specific to a medical condition.**

I have used PRESCRIBED medicinal cannabis to manage symptoms of: (Choose up to three symptoms).

- ☐ Anxiety
- ☐ Agitation
- ☐ Breathing (respiratory) problems
- ☐ Cognitive problems (e.g. problems with memory, concentration)
- ☐ Depression
- ☐ Diarrhoea/constipation
- ☐ Drug withdrawal
- ☐ Headache
- ☐ Inflammation
- ☐ Muscle spasms
- ☐ Nausea vomiting
- ☐ Occular pressure
- ☐ Pain
- ☐ Poor appetite/weight loss
- ☐ Psychosis
- ☐ Seizures
- ☐ Skin
- ☐ Sleep
- ☐ Other symptom

(select all that apply)

Please rate how your anxiety has changed since using prescribed medicinal cannabis:

- ◯ Very much better
- ◯ Much better
- ◯ A little better
- ◯ No change
- ◯ A little worse
- ◯ Much worse
- ◯ Very much worse

(select one option)

Please rate how your agitation has changed since using prescribed medicinal cannabis:

- ◯ Very much better
- ◯ Much better
- ◯ A little better
- ◯ No change
- ◯ A little worse
- ◯ Much worse
- ◯ Very much worse

(select one option)

Please rate how your respiratory symptoms you have changed since using prescribed medicinal cannabis:

- ◯ Very much better
- ◯ Much better
- ◯ A little better
- ◯ No change
- ◯ A little worse
- ◯ Much worse
- ◯ Very much worse

(select one option)

| | |
|---|---|
| Please rate how your cognitive problems have changed since using prescribed medicinal cannabis | ◯ Very much better<br>◯ Much better<br>◯ A little better<br>◯ No change<br>◯ A little worse<br>◯ Much worse<br>◯ Very much worse<br>(select one option) |
| Please rate how your depression has changed since using prescribed medicinal cannabis: | ◯ Very much better<br>◯ Much better<br>◯ A little better<br>◯ No change<br>◯ A little worse<br>◯ Much worse<br>◯ Very much worse<br>(select one option) |
| Please rate how your diarrhoea/constipation has changed since using prescribed medicinal cannabis: | ◯ Very much better<br>◯ Much better<br>◯ A little better<br>◯ No change<br>◯ A little worse<br>◯ Much worse<br>◯ Very much worse<br>(select one option) |
| Please rate how your symptoms of drug withdrawal have changed since using prescribed medicinal cannabis: | ◯ Very much better<br>◯ Much better<br>◯ A little better<br>◯ No change<br>◯ A little worse<br>◯ Much worse<br>◯ Very much worse<br>(select one option) |
| Please rate how your headaches have changed since using  prescribed medicinal cannabis: | ◯ Very much better<br>◯ Much better<br>◯ A little better<br>◯ No change<br>◯ A little worse<br>◯ Much worse<br>◯ Very much worse<br>(select one option) |
| Please rate how your Inflammation has changed since using prescribed medicinal cannabis: | ◯ Very much better<br>◯ Much better<br>◯ A little better<br>◯ No change<br>◯ A little worse<br>◯ Much worse<br>◯ Very much worse<br>(select one option) |

| | |
|---|---|
| Please rate how your muscle spasms have changed since using  prescribed medicinal cannabis: | ◯ Very much better<br>◯ Much better<br>◯ A little better<br>◯ No change<br>◯ A little worse<br>◯ Much worse<br>◯ Very much worse<br>(select one option) |
| Please rate how your nausea/vomiting has changed using  prescribed medicinal cannabis: | ◯ Very much better<br>◯ Much better<br>◯ A little better<br>◯ No change<br>◯ A little worse<br>◯ Much worse<br>◯ Very much worse<br>(select one option) |
| Please rate how your symptoms of occular pressure have changed since using  prescribed medicinal cannabis: | ◯ Very much better<br>◯ Much better<br>◯ A little better<br>◯ No change<br>◯ A little worse<br>◯ Much worse<br>◯ Very much worse<br>(select one option) |
| Please rate how your pain has changed since using prescribed medicinal cannabis: | ◯ Very much better<br>◯ Much better<br>◯ A little better<br>◯ No change<br>◯ A little worse<br>◯ Much worse<br>◯ Very much worse<br>(select one option) |
| Please rate how your appetite/weight has changed since starting  prescribed medicinal cannabis: | ◯ Very much better<br>◯ Much better<br>◯ A little better<br>◯ No change<br>◯ A little worse<br>◯ Much worse<br>◯ Very much worse<br>(select one option) |
| Please rate how your psychosis has changed since using prescribed medicinal cannabis: | ◯ Very much better<br>◯ Much better<br>◯ A little better<br>◯ No change<br>◯ A little worse<br>◯ Much worse<br>◯ Very much worse<br>(select one option) |

| | |
|---|---|
| Please rate how your seizures have changed since using prescribed medicinal cannabis: | ◯ Very much better<br>◯ Much better<br>◯ A little better<br>◯ No change<br>◯ A little worse<br>◯ Much worse<br>◯ Very much worse<br>(select one option) |
| Please rate how your skin complaints have changed since using prescribed medicinal cannabis: | ◯ Very much better<br>◯ Much better<br>◯ A little better<br>◯ No change<br>◯ A little worse<br>◯ Much worse<br>◯ Very much worse<br>(select one option) |
| Please rate how your sleep problems have changed since using prescribed medicinal cannabis: | ◯ Very much better<br>◯ Much better<br>◯ A little better<br>◯ No change<br>◯ A little worse<br>◯ Much worse<br>◯ Very much worse<br>(select one option) |
| Please describe any other symptoms you have used prescribed medicinal cannabis to treat/cure | ______________________________<br>(Please indicate NO MORE than three other symptoms) |
| For your MAIN other symptom (choose one), how has this symptom changed since starting prescribed medicinal cannabis? | ◯ Very much better<br>◯ Much better<br>◯ A little better<br>◯ No change<br>◯ A little worse<br>◯ Much worse<br>◯ Very much worse<br>(select one option) |

**The following questions ask about the type of PRESCRIBED medicinal cannabis you use.**

What type of medicinal cannabis you are MAINLY PRESCRIBED?

- ◯ THC only
- ◯ Mainly THC and small amount of other cannabinoids
- ◯ Approximately equal amounts of THC and CBD
- ◯ Mainly CBD and small amounts of other cannabinoids (e.g. THC)
- ◯ CBD only
- ◯ Unsure/Do not know
- ◯ It varies between batches
- ◯ Other

(select one option)

---

What other type of cannabinoid is in the medicinal cannabis you are MAINLY PRESCRIBED?

__________________________________
(please describe)

---

Do you feel that the quality or composition of your prescribed medicinal cannabis is consistent over time?

- ◯ Yes
- ◯ No
- ◯ Unsure/Don't know

(select one option)

---

Do you worry about accessing your preferred source of legal (prescribed) cannabis?

- ◯ Very much so
- ◯ Somewhat
- ◯ No opinion
- ◯ Not especially
- ◯ Not at all

(select one option)

---

In the last 28 days have you ever been unable to access your preferred source of legal (prescribed) medicinal cannabis to treat your health concerns?

- ◯ Yes
- ◯ No

---

Are you concerned about the possibility of contaminants in your prescribed medicinal cannabis? (e.g. heavy metals, toxins, pesticides, bacteria/fungi/mould)

- ◯ Not at all
- ◯ A little
- ◯ Not sure
- ◯ Quite a bit
- ◯ A great deal

(select one option)

---

What is the MAIN way you consume PRESCRIBED medicinal cannabis?

- ◯ Oral (tablet or capsule)
- ◯ Oral edibles (cake, cookie etc.)
- ◯ Oral liquid (oil, tincture, etc.)
- ◯ Oral spray (mouth or oromucosal)
- ◯ Fresh juice
- ◯ Nasal application (through nose)
- ◯ Rolled into a joint
- ◯ Pipe (plastic, metal, glass)
- ◯ Water pipe/ Bong
- ◯ Vaporiser
- ◯ Suppository
- ◯ Dabbing or spotting
- ◯ Topical (cream or patch applied to the skin)
- ◯ Other

(select one option)

---

What other way have you consumed prescribed medicinal cannabis?

__________________________________
(please describe one other way of consuming cannabis)

When using your usual dose of prescribed medicinal cannabis how long does it take for you to feel ANY effect? Please indicate the number of minutes as a whole number.

__________________________________
(please enter a number of MINUTES (1 hr = 60))

When using your usual dose of prescribed medicinal cannabis how long does it take to achieve its MAXIMUM effect (e.g. relief from symptoms)? Please indicate the number of minutes as a whole number.

__________________________________
(please enter a number of MINUTES (1 hr = 60))

__________________________________

The time you entered as the time it takes for you to feel the MAXIMUM effect of cannabis is lower than the time you entered as the time it takes you to feel ANY effect. This does not make sense. Please adjust your answers accordingly.

When using your usual dose of prescribed medicinal cannabis how long would you estimate its effects last for (i.e. how long before you no longer feel any effects)?

__________________________________
(please enter a number of MINUTES (1 hr = 60min))

__________________________________

The time you entered as the time it takes until you NO LONGER FEEL ANY EFFECTS is less than the time you entered as the time it takes for you to feel ANY effect. This does not make sense. Please adjust your answers to these questions accordingly.

__________________________________

The time you entered as the time it takes until you NO LONGER FEEL ANY EFFECTS is less than the time you entered as the time it takes for you to feel MAXIMUM effects. This does not make sense. Please adjust your answers to these questions accordingly.

**The following questions refer to your use of ILLICIT (NON-PRESCRIBED) medicinal cannabis. Illicit (non-prescribed) medicinal cannabis is not obtained from a pharmacy, but from other sources (dealer, friends or family, online vendor, grow your own etc.)**

| | |
|---|---|
| In the last 12 MONTHS, in which of the following ways have you accessed your illicit (non-prescribed) medicinal cannabis supply?<br><br>You may select more than one option<br><br>Note: We recognise that "I grow my own" is a legal practice in the ACT. | ☐ I grow my own<br>☐ From friends or family<br>☐ From a recreational dealer<br>☐ From a medicinal cannabis supplier (not prescribed by a medicinal practitioner)<br>☐ From a 'club' or 'co-operative' outlet<br>☐ From an online supplier<br>☐ From an overseas supplier<br>☐ Other<br>(select all that apply) |
| Which other ways have you used to access illicit (non-prescribed) medicinal cannabis? | ______________________________<br>(please describe) |
| In the last 12 MONTHS, what is the MAIN way you accessed your illicit (non-prescribed) medicinal cannabis?<br><br>Select one option only<br><br>Note: We recognise that "I grow my own" is a legal practice in the ACT. | ○ I grow my own<br>○ From friends or family<br>○ From a recreational dealer<br>○ From a medicinal cannabis supplier (not prescribed by a medical practitioner)<br>○ From a 'club' or 'co-operative' outlet<br>○ From an online supplier<br>○ From an overseas supplier<br>○ Other |
| What is the other MAIN way you have used to access medicinal cannabis? | ______________________________<br>(Please provide a brief description) |
| The following question asks about your illicit (non-prescribed) medicinal cannabis use.<br><br>In the last 12 MONTHS, in which of the following ways have you accessed your medicinal cannabis supply?<br><br>You may select more than one option<br><br>Note: We recognise that "I grow my own" is a legal practice in the ACT. | ☐ I grow my own<br>☐ From friends or family<br>☐ From a recreational dealer<br>☐ From a medicinal cannabis supplier (not prescribed by a medical practitioner)<br>☐ From a 'club' or 'co-operative' outlet<br>☐ From an online supplier<br>☐ From an overseas supplier<br>☐ Other<br>(select all that apply) |
| Which other ways have you used to access illicit (non-prescribed) medicinal cannabis? | ______________________________<br>(please describe) |
| The following question asks about your illicit (non-prescribed) medicinal cannabis use.<br><br>In the last 12 MONTHS, what is the MAIN way you accessed your medicinal cannabis?<br><br>Select one option only<br><br>Note: We recognise that "I grow my own" is a legal practice in the ACT. | ○ I grow my own<br>○ From friends or family<br>○ From a recreational dealer<br>○ From a medicinal cannabis supplier (not prescribed by a medical practitioner)<br>○ From a 'club' or 'co-operative' outlet<br>○ From an online supplier<br>○ From an overseas supplier<br>○ Other |

| | |
|---|---|
| What is the other MAIN way you have used to access illicit (non-prescribed) medicinal cannabis? | __________________________________ (Please provide a brief description) |
| Have you used ILLICIT medicinal cannabis to treat a condition from any of the following groups?<br><br>You may select more than one option | ☐ Cancer<br>☐ Gastro-intestinal<br>☐ Mental health and/or substance use<br>☐ Neurological<br>☐ Pain<br>☐ Sleep<br>☐ Other<br>(select all that apply) |
| Please indicate which of the following cancer conditions you have used ILLICIT medicinal cannabis to treat. | ☐ Blood cancers (leukaemia, lymphoma, myeloma)<br>☐ Brain cancers (glioblastoma, neuroblastoma, mesothelioma)<br>☐ Breast cancer<br>☐ Gastrointestinal cancer (bowel, colon, stomach, pancreatic)<br>☐ Lung cancer<br>☐ Reproductive cancers (prostate, cervical, uterine, testicular, ovarian)<br>☐ Skin cancers (melanoma)<br>☐ Other forms of cancer<br>(select all that apply (limit of three)) |
| What other type of cancer have you used illicit medicinal cannabis to treat? | __________________________________ |
| Please indicate which of the following gastro-intestinal conditions you have used ILLICIT medicinal cannabis to treat. | ☐ Crohn's disease<br>☐ Irritable bowel syndrome<br>☐ Ulcerative collitis<br>☐ Other gastro-intestinal condition<br>(select all that apply (limit of three)) |
| What other type of gastro-intestinal conditions have you used illicit medicinal cannabis to treat? | __________________________________ |
| Please indicate which of the following mental health conditions you have used ILLICIT medicinal cannabis to treat. | ☐ Addiction (Cannabis)<br>☐ Addiction (Other drugs: including alcohol, opioid, amphetamine)<br>☐ Anxiety Disorder (e.g. generalised anxiety, panic disorder, OCD)<br>☐ Attention Deficit disorder (ADHD)<br>☐ Depression<br>☐ Eating disorders (e.g. anorexia / bulimia/ obesity)<br>☐ Manic Disorder (Bipolar affective disorder)<br>☐ Post-Traumatic Stress Disorder<br>☐ Schizophrenia or other psychosis<br>☐ Other mental health condition<br>(select all that apply (limit of three)) |
| What other type of mental health condition do you use illicit medicinal cannabis to treat? | __________________________________ |

| | |
|---|---|
| Please indicate which of the following neurological conditions you use ILLICIT medicinal cannabis to treat.<br><br>Please note: for the purposes of this survey fibromyalgia, neuropathy, and mental illnesses (e.g. depression, anxiety, ADHD, Schizophrenia) are NOT considered to be neuorological disorders. If you clicked on this category in order to indicate one of these conditions you will find fibromyalgia and neuropathy under pain conditions and depression, anxiety, ADHD, and Schizophrenia under mental health and/or substance use conditions. | ☐ Amyotrophic Lateral Sclerosis<br>☐ Autism<br>☐ Dementia (including Alzheimer's)<br>☐ Epilepsy/ Seizure disorder<br>☐ Glaucoma<br>☐ Huntington's disease<br>☐ Multiple Sclerosis<br>☐ Parkinson's Disease<br>☐ Tourette's Syndrome<br>☐ Other neurological condition<br>(select all that apply (limit of three)) |
| What other type of neurological conditions have you used illicit medicinal cannabis to treat? | __________________________________<br>(please describe) |
| Please indicate which of the following pain conditions you have used ILLICIT medicinal cannabis to treat | ☐ Arthritis (including rheumatoid or osteoarthritis<br>☐ Back pain<br>☐ Cancer-related pain<br>☐ Fibromyalgia<br>☐ Gynaecological pain<br>☐ Headaches (including migraines)<br>☐ Menstrual pain<br>☐ Neck pain<br>☐ Neuropathy (nerve pain)<br>☐ Spinal cord injury<br>☐ Other type of chronic, non-cancer pain not described above<br>(select all that apply (limit of three)) |
| What other type of pain disorder do you use illicit medicinal cannabis to treat? | __________________________________<br>(please describe) |
| Please indicate which of the following sleep conditions you have used ILLICIT medicinal cannabis to treat | ☐ Circadian rhythm sleep disorder (e.g. shift work type)<br>☐ Insomnia (any type)<br>☐ Narcolepsy or other hypersomnia<br>☐ Parasomnias (e.g. sleep walking, nightmares)<br>☐ Sleep apnoea or other sleep-related breathing disorder<br>☐ Sleep-related movement disorder (e.g. restless leg syndrome)<br>☐ Other sleep disorder<br>(select all that apply (limit of three)) |
| What other type of sleep disorder do you use illicit medicinal cannabis to treat? | __________________________________<br>(please describe) |

| | |
|---|---|
| Please indicate which of the following other conditions you use ILLICIT medicinal cannabis to treat | ☐ Auto-Immune condition (e.g. SLE, Chronic Fatigue disorder)<br>☐ Cardiovascular condition (e.g. poor circulation, ischemic heart disease)<br>☐ Diabetes Mellitus<br>☐ Gynaecological condition (e.g. endometriosis, PMS)<br>☐ AIDS/HIV<br>☐ Infectious disease (e.g. viral hepatitis)<br>☐ Respiratory disease (e.g. asthma, cystic fibrosis)<br>☐ Skin condition (e.g. eczema, psoriasis, dermatitis)<br>☐ Other condition<br>(select all that apply (limit of five)) |
| What other conditions have you used illicit medicinal cannabis to treat? | ______________________________<br>(please describe) |

**The following questions ask about the MAIN health condition for which you have used ILLICIT (non-prescribed) cannabis for medical purposes, and how this condition changed. The main health condition I use illicit medicinal cannabis for is:**

| | MAIN condition |
|---|---|
| Addiction (Cannabis) | ○ |
| Addiction (Other drugs: including alcohol, opioid, amphetamine) | ○ |
| Amyotrophic Lateral Sclerosis | ○ |
| Anxiety Disorder (e.g. generalised anxiety, panic disorder, OCD) | ○ |
| Arthritis (including rheumatoid or osteoarthritis | ○ |
| Attention Deficit disorder (ADHD) | ○ |
| Autism | ○ |
| Auto-Immune condition (e.g. SLE, chronic fatigue disorder) | ○ |
| Back pain | ○ |
| Blood cancers (leukaemia, lymphoma) | ○ |
| Brain cancers (glioblastoma, neuroblastoma, mesothelioma) | ○ |
| Breast cancer | ○ |
| Cancer-related pain | ○ |
| Cardiovascular condition (e.g. poor circulation, ischemic heart disease) | ○ |
| Circadian rhythm sleep disorder (e.g. shift work type) | ○ |
| Crohn's disease | ○ |
| Dementia (including Alzheimer's) | ○ |
| Depression | ○ |
| Diabetes Mellitus | ○ |
| Eating disorders (e.g. anorexia / bulimia/ obesity) | ○ |
| Epilepsy/ Seizure disorder | ○ |
| Fibromyalgia | ○ |
| Gastrointestinal cancer (bowel, colon, stomach) | ○ |
| Glaucoma | ○ |

| | |
|---|---|
| Gynaecological condition (e.g. endometriosis, PMT) | ◯ |
| Headaches (including migraines) | ◯ |
| Huntington's disease | ◯ |
| Infectious disease (e.g. AIDS/HIV, viral hepatitis) | ◯ |
| Insomnia (any type) | ◯ |
| Irritable bowel syndrome | ◯ |
| Lung cancer | ◯ |
| Manic Disorder (Bipolar affective disorder) | ◯ |
| Menstrual pain | ◯ |
| Multiple Sclerosis | ◯ |
| Narcolepsy or other hypersomnia | ◯ |
| Neuropathy (nerve pain) | ◯ |
| [condition_cancer_other_2] | ◯ |
| [condition_gastro_other_2] | ◯ |
| [condition_mentalh_other_2] | ◯ |
| [condition_neuro_other_2] | ◯ |
| [condition_sleep_other_2] | ◯ |
| [condition_pain_other_2] | ◯ |
| Parasomnias (e.g. sleep walking, nightmares) | ◯ |
| Post-Traumatic Stress Disorder | ◯ |
| Reproductive cancers (cervical, uterine, testicular, ovarian) | ◯ |
| Respiratory disease (e.g. asthma, cystic fibrosis) | ◯ |
| Schizophrenia or other psychosis | ◯ |
| Skin cancers (melanoma) | ◯ |
| Skin condition (e.g. eczema, psoriasis, dermatitis) | ◯ |
| Sleep apnoea or other sleep-related breathing disorder | ◯ |
| Sleep-related movement disorder (e.g. restless leg syndrome) | ◯ |
| Spinal cord injury | ◯ |
| Tourette's Syndrome | ◯ |
| Ulcerative collitis | ◯ |
| [condition_other_other_2] | ◯ |
| Neck pain | ◯ |

| | |
|---|---|
| Please check the option that best describes how your MAIN health condition is now compared with how it was before using ILLICIT medicinal cannabis? | ◯ Very much better<br>◯ Much better<br>◯ A little better<br>◯ No change<br>◯ A little worse<br>◯ Much worse<br>◯ Very much worse<br>(select one option) |

**Please indicate which of the following SYMPTOMS you have used ILLICIT medicinal cannabis to manage in the past 12 months Please keep in mind that a medical condition refers to an injury, illness, or disease that affects your physical and/or mental health. In contrast, symptoms are what people experience (e.g. nausea, pain) and are often not specific to a medical condition.**

I have used ILLICIT medicinal cannabis to manage symptoms of: (Choose up to three symptoms).

- ☐ Anxiety
- ☐ Agitation
- ☐ Breathing (respiratory) problems
- ☐ Cognitive problems (e.g. problems with memory, concentration)
- ☐ Depression
- ☐ Diarrhoea/constipation
- ☐ Drug withdrawal
- ☐ Headache
- ☐ Inflammation
- ☐ Muscle spasms
- ☐ Nausea vomiting
- ☐ Occular pressure
- ☐ Pain
- ☐ Poor appetite/weight loss
- ☐ Psychosis
- ☐ Seizures
- ☐ Skin
- ☐ Sleep
- ☐ Other symptom

(select all that apply)

Please rate how your anxiety has changed since using illicit medicinal cannabis:

- ◯ Very much better
- ◯ Much better
- ◯ A little better
- ◯ No change
- ◯ A little worse
- ◯ Much worse
- ◯ Very much worse

(select one option)

Please rate how your agitation has changed since using illicit medicinal cannabis:

- ◯ Very much better
- ◯ Much better
- ◯ A little better
- ◯ No change
- ◯ A little worse
- ◯ Much worse
- ◯ Very much worse

(select one option)

Please rate how your respiratory symptoms you have changed since using illicit medicinal cannabis:

- ◯ Very much better
- ◯ Much better
- ◯ A little better
- ◯ No change
- ◯ A little worse
- ◯ Much worse
- ◯ Very much worse

(select one option)

| | |
|---|---|
| Please rate how your cognitive problems have changed since using illicit medicinal cannabis | ◯ Very much better<br>◯ Much better<br>◯ A little better<br>◯ No change<br>◯ A little worse<br>◯ Much worse<br>◯ Very much worse<br>(select one option) |
| Please rate how your depression has changed since using illicit medicinal cannabis: | ◯ Very much better<br>◯ Much better<br>◯ A little better<br>◯ No change<br>◯ A little worse<br>◯ Much worse<br>◯ Very much worse<br>(select one option) |
| Please rate how your diarrhoea/constipation has changed since using illicit medicinal cannabis: | ◯ Very much better<br>◯ Much better<br>◯ A little better<br>◯ No change<br>◯ A little worse<br>◯ Much worse<br>◯ Very much worse<br>(select one option) |
| Please rate how your symptoms of drug withdrawal have changed since using illicit medicinal cannabis: | ◯ Very much better<br>◯ Much better<br>◯ A little better<br>◯ No change<br>◯ A little worse<br>◯ Much worse<br>◯ Very much worse<br>(select one option) |
| Please rate how your headaches have changed since using illicit medicinal cannabis: | ◯ Very much better<br>◯ Much better<br>◯ A little better<br>◯ No change<br>◯ A little worse<br>◯ Much worse<br>◯ Very much worse<br>(select one option) |
| Please rate how your Inflammation has changed since using illicit medicinal cannabis: | ◯ Very much better<br>◯ Much better<br>◯ A little better<br>◯ No change<br>◯ A little worse<br>◯ Much worse<br>◯ Very much worse<br>(select one option) |

| | |
|---|---|
| Please rate how your muscle spasms have changed since using illicit medicinal cannabis: | ◯ Very much better<br>◯ Much better<br>◯ A little better<br>◯ No change<br>◯ A little worse<br>◯ Much worse<br>◯ Very much worse<br>(select one option) |
| Please rate how your nausea/vomiting has changed using illicit medicinal cannabis: | ◯ Very much better<br>◯ Much better<br>◯ A little better<br>◯ No change<br>◯ A little worse<br>◯ Much worse<br>◯ Very much worse<br>(select one option) |
| Please rate how your symptoms of occular pressure have changed since using illicit medicinal cannabis: | ◯ Very much better<br>◯ Much better<br>◯ A little better<br>◯ No change<br>◯ A little worse<br>◯ Much worse<br>◯ Very much worse<br>(select one option) |
| Please rate how your pain has changed since using illicit medicinal cannabis: | ◯ Very much better<br>◯ Much better<br>◯ A little better<br>◯ No change<br>◯ A little worse<br>◯ Much worse<br>◯ Very much worse<br>(select one option) |
| Please rate how your appetite/weight has changed since starting illicit medicinal cannabis: | ◯ Very much better<br>◯ Much better<br>◯ A little better<br>◯ No change<br>◯ A little worse<br>◯ Much worse<br>◯ Very much worse<br>(select one option) |
| Please rate how your Psychosis has changed since using illicit medicinal cannabis: | ◯ Very much better<br>◯ Much better<br>◯ A little better<br>◯ No change<br>◯ A little worse<br>◯ Much worse<br>◯ Very much worse<br>(select one option) |

| | |
|---|---|
| Please rate how your seizures have changed since using illicit medicinal cannabis: | ◯ Very much better<br>◯ Much better<br>◯ A little better<br>◯ No change<br>◯ A little worse<br>◯ Much worse<br>◯ Very much worse<br>(select one option) |
| Please rate how your skin complaints have changed since using illicit medicinal cannabis: | ◯ Very much better<br>◯ Much better<br>◯ A little better<br>◯ No change<br>◯ A little worse<br>◯ Much worse<br>◯ Very much worse<br>(select one option) |
| Please rate how your sleep problems have changed since using illicit medicinal cannabis: | ◯ Very much better<br>◯ Much better<br>◯ A little better<br>◯ No change<br>◯ A little worse<br>◯ Much worse<br>◯ Very much worse<br>(select one option) |
| Please describe any other symptoms you have used illicit medicinal cannabis to treat/cure | ________________________________<br>(Please indicate NO MORE than three other symptoms) |
| For your MAIN other symptom (choose one), how has this symptom changed since starting illicit medicinal cannabis? | ◯ Very much better<br>◯ Much better<br>◯ A little better<br>◯ No change<br>◯ A little worse<br>◯ Much worse<br>◯ Very much worse<br>(select one option) |

**The following questions ask about the type of ILLICIT (NON-PRESCRIBED) medicinal cannabis you have used in the past 12 months.**

| | |
|---|---|
| What type of illicit (non-prescribed) medicinal cannabis do you MAINLY use? | ◯ THC only<br>◯ Mainly THC and small amount of other cannabinoids<br>◯ Approximately equal amounts of THC and CBD<br>◯ Mainly CBD and small amounts of other cannabinoids (e.g. THC)<br>◯ CBD only<br>◯ Unsure/Do not know<br>◯ It varies between batches<br>◯ Other<br>(select one option) |
| What other type of illicit (non-prescribed) cannabinoid is in the medicinal cannabis you use? | __________________________________<br>(please describe) |
| Do you feel that the quality or composition of your illicit (non-prescribed) medicinal cannabis is consistent over time? | ◯ Yes<br>◯ No<br>◯ Unsure/Don't know<br>(select one option) |
| Do you worry about accessing your preferred source of illicit (non-prescribed) cannabis? | ◯ Very much so<br>◯ Somewhat<br>◯ No opinion<br>◯ Not especially<br>◯ Not at all<br>(select one option) |
| In the last 28 days have you ever been unable to access your preferred source of illicit (non-prescribed) medicinal cannabis to treat your health concerns? | ◯ Yes<br>◯ No |
| Are you concerned about the possibility of contaminants in your illicit (non-prescribed) cannabis? (e.g. heavy metals, toxins, pesticides, bacteria/fungi/mould) | ◯ Not at all<br>◯ A little<br>◯ Not sure<br>◯ Quite a bit<br>◯ A great deal<br>(select one option) |
| What is the MAIN way you consume your illicit (non-prescribed) cannabis? | ◯ Oral (tablet or capsule)<br>◯ Oral edibles (cake, cookie etc.)<br>◯ Oral liquid (oil, tincture, etc.)<br>◯ Oral spray (mouth or oromucosal)<br>◯ Fresh juice<br>◯ Nasal application (through nose)<br>◯ Rolled into a joint<br>◯ Pipe (plastic, metal, glass)<br>◯ Water pipe/ Bong<br>◯ Vaporiser<br>◯ Suppository<br>◯ Dabbing or spotting<br>◯ Topical (cream or patch applied to the skin)<br>◯ Other<br>(select one option) |

What other way have you consumed illicit (non-prescribed) cannabis?

__________________________________
(please describe one other way of consuming cannabis)

When using your usual dose of illicit (non-prescribed) medicinal cannabis how long does it take for you to feel ANY effect?

__________________________________
(please enter a number of MINUTES (1 hr = 60min))

When using your usual dose of illicit (non-prescribed) medicinal cannabis how long does it take to achieve its MAXIMUM effect (e.g. relief from symptoms)?

__________________________________
(please enter a number of MINUTES (1 hr = 60min))

__________________________________

The time you entered as the time it takes for you to feel the MAXIMUM effect of cannabis is lower than the time you entered as the time it takes you to feel ANY effect. This does not make sense. Please adjust your answers accordingly.

When using your usual dose of illicit (non-prescribed) medicinal cannabis how long would you estimate its effects last for (i.e. how long before you no longer feel any effects)?

__________________________________
(please enter a number of MINUTES (1 hr = 60min))

__________________________________

The time you entered as the time it takes until you NO LONGER FEEL ANY EFFECTS is less than the time you entered as the time it takes for you to feel ANY effect. This does not make sense. Please adjust your answers to these questions accordingly.

__________________________________

The time you entered as the time it takes until you NO LONGER FEEL ANY EFFECTS is less than the time you entered as the time it takes for you to feel MAXIMUM effects. This does not make sense. Please adjust your answers to these questions accordingly.

## SECTION 4

## PATTERNS OF CANNABIS USE, IN GENERAL

How old were you when you first tried cannabis for ANY reason?

__________________________________
(years old (in numbers))

How old were you when you first tried cannabis for MEDICAL purposes?

__________________________________
(years old (in numbers))

__________________________________

The age you entered as the age you first used cannabis for MEDICAL reasons is lower than the age you first tried cannabis for ANY reason. This does not make sense. Please think carefully and adjust your answers accordingly.

How old were you when you first started using cannabis REGULARLY for ANY reason? (at least once per week for a period of three months or longer).

If you have never used cannabis REGULARLY please enter '0'

__________________________________
(years old (in numbers). If you have never used cannabis regularly please enter '0')

__________________________________

The age you entered as the age you first used cannabis REGULARLY is lower than the age you entered as the age you FIRST TRIED cannabis. This does not make sense. Please think carefully and adjust your answers accordingly.

How old were you when you first started using cannabis REGULARLY for MEDICAL purposes? (at least once per week for a period of three months or longer).

If you have never used cannabis REGULARLY for MEDICAL reasons please enter '0'

__________________________________
(years old (in numbers). If you have never used cannabis regularly for medical reasons please enter '0')

__________________________________

__________________________________

The age you entered as the age you first used cannabis REGULARLY for MEDICAL purposes is lower than the age you entered as the age you FIRST used cannabis for MEDICAL purposes. This does not make sense. Please think carefully and adjust your answers accordingly.

__________________________________

The age you entered as the age you first used cannabis REGULARLY for MEDICAL reasons is lower than the age you entered as the age you first used cannabis REGULARLY for ANY reason (medical reasons are included in any reason). This does not make sense. Please think carefully and adjust your answers to these questions accordingly.

__________________________________

In the previous question you entered '0', indicating that you had NEVER used cannabis for ANY reason REGULARLY, yet you have given a value greater than 0 for the age you began using cannabis REGULARLY for MEDICAL reasons. This does not make sense. Please think carefully and adjust your answers to these questions accordingly.

Which of the following best describes your use of cannabis before you used it for medical reasons?

- ◯ I had not used cannabis before using it for medical reasons
- ◯ I had used cannabis for non-medical reasons but had quit for a year or more before taking up cannabis use for medical reasons.
- ◯ I was using cannabis for non-medical purposes at the time when I started using it medically.

(select one option)

Please estimate the time, in months, between your last non-medical use of cannabis and starting medicinal cannabis

________________________________
(number of months (e.g. if one year and two months enter '14', if < 1 month e.g. 2 weeks = 0.5, 3 weeks = 0.75))

In the last 28 days, please estimate how many days you used cannabis for ANY reason:

________________________________
(enter (0-28))

On an average day that you use cannabis for ANY reason, on how many occasions do you usually use cannabis (i.e. how many times a day)?

________________________________
((max 20))

In the last 28 days, please estimate how many days you used cannabis for MEDICAL reasons:

________________________________
(enter (0-28))

________________________________

The value you entered for number of days using cannabis for MEDICAL reasons is greater than your entry for the number of days using cannabis for ANY reason (i.e. including medical). This does not make sense (days' use MEDICAL can only ever be equal to or less than days' use for ANY reason). Please adjust your answers to these questions accordingly.

On an average day that you use cannabis for MEDICAL reasons, on how many occasions do you usually use cannabis (how many times a day)?

________________________________
((max 20))

________________________________

The value you entered for number of times per day you use cannabis for MEDICAL reasons is greater than the value you entered for number of times per day for ANY reason. This does not make sense (times used per day for MEDICAL reasons must always be equal to or less than times per day for ANY reason). Please adjust your answers to these questions accordingly.

On the days that you use cannabis, please indicate the percentage of use you consider to be MEDICAL, as a proportion of your total use for ANY reason:

0% 50% 100%

*(Place a mark on the scale above)*

| | |
|---|---|
| Did your cannabis use change due to COVID-19? | ◯ Yes, my proportion of use that was medical increased<br>◯ Yes, my proportion of use that was non-medical increased<br>◯ No, my cannabis use did not change |
| In the last 12 months have you had a break of one month or more from using cannabis for medical reasons? | ◯ Yes<br>◯ No |
| Did you take a break after using cannabis heavily or for a prolonged time? i.e. usually daily or almost daily use over a period of at least a few months. | ◯ Yes<br>◯ No |
| Why did you stop using medicinal cannabis? | ☐ Unable to find supply<br>☐ Could not afford it<br>☐ Did not like the side effects (including the 'high')<br>☐ It did not work<br>☐ I no longer suffered from my health complaint<br>☐ I wanted a break from cannabis use<br>☐ Other<br>(select all that apply) |
| Please tell us the other reason you stopped using medicinal cannabis | ______________________________<br>(please describe) |
| Please estimate the cost of your medicinal cannabis on an average WEEKLY basis in Australian dollars.<br><br>If you do not purchase your medicinal cannabis please enter zero (0). | ______________________________<br>(amount per week in dollars) |

**EXPERIENCES WHEN STOPPING CANNABIS USE**

**Within a week of stopping cannabis use that has been heavy or prolonged (i.e. usually daily or almost daily use over a period of at least a few months) I develop...**

| | Yes | No |
|---|---|---|
| Irritability, anger or aggression | ○ | ○ |
| Nervousness or anxiety | ○ | ○ |
| Sleep difficulty (e.g., insomnia, disturbing dreams) | ○ | ○ |
| Decreased appetite or weight loss | ○ | ○ |
| Restlessness | ○ | ○ |
| Depressed mood | ○ | ○ |
| Significant distress from at least one of the following: abdominal pain, shakiness/tremors, sweating, fever, chills or headache | ○ | ○ |
| I take cannabis (or a closely related substance) to relieve or avoid withdrawal symptoms | ○ | ○ |

## SECTION 5

## Medicinal Cannabis, Health Care and Social Issues

| Question | Response |
| --- | --- |
| Which of the following MOST informed your decision to use medicinal cannabis for your health condition? | ◯ Discovered benefits on my own (i.e. was using cannabis and noticed my symptoms improved)<br>◯ Social media (e.g. Facebook, Reddit, Instagram)<br>◯ Conventional media (e.g. television, radio, newspaper, magazine)<br>◯ Conventional healthcare provider (e.g. GP, medical specialist, pharmacist, nurse, counsellor)<br>◯ Alternative healthcare practitioner (e.g. herbalist, naturopath)<br>◯ Friend or family member<br>◯ Personal research (e.g. journal articles, books, google scholar)<br>◯ Consumer group focused on a particular condition (e.g. epilepsy, chronic pain, mental health)<br>◯ Medicinal cannabis advocacy group (e.g. United in Compassion, Hemp embassy)<br>◯ Other<br>(select one option) |
| What other source most informed your decision to use medicinal cannabis for your condition? | __________________________________ |
| Have you discussed your medicinal cannabis use with any of your healthcare providers? | ◯ Yes - with most of my healthcare providers<br>◯ Yes - but only with a small number of my healthcare providers<br>◯ No - not with any of my healthcare providers<br>(select one option) |
| Please indicate which healthcare providers you have discussed your medicinal cannabis use with | ☐ General Practitioner (GP, including GP at private cannabis clinic)<br>☐ Specialist medical practitioner (e.g. pain specialist, neurologist, oncologist, psychiatrist, specialist at private cannabis clinic)<br>☐ Counsellor/psychologist<br>☐ Nurse<br>☐ Pharmacist<br>☐ Physiotherapist<br>☐ Alternative therapist (e.g. acupuncturist, herbalist)<br>☐ Other<br>(select all that apply) |
| Please tell us what other kind of healthcare provider you have discussed your medicinal cannabis use with. | __________________________________<br>(please describe) |
| Which of the following best describes your healthcare provider's general attitude to your use of medicinal cannabis? | ◯ All are supportive of my medicinal cannabis use<br>◯ Most (but not all) are supportive of my medicinal cannabis use<br>◯ Uncertain<br>◯ Most (but not all) do not support my medicinal cannabis use<br>◯ All do not support my medicinal cannabis use<br>(select one option) |

| | |
|---|---|
| Did you feel comfortable disclosing the details of your medicinal cannabis use to your medical practitioners? | ◯ Yes - to most of my medical practitioners<br>◯ Yes - but only to one or a few of my medical practitioners<br>◯ No |
| Do you feel comfortable disclosing the details of your medicinal cannabis use with close friends/family? | ◯ Yes - to most of my friends/family<br>◯ Yes - but only to some close friends/family members<br>◯ No<br>(select one option) |
| Which of the following best describes your family and friends' general attitude to your use of medicinal cannabis | ◯ All are supportive of my medicinal cannabis use<br>◯ Most (but not all) are supportive of my medicinal cannabis use<br>◯ Uncertain<br>◯ Most (but not all) do not support my medicinal cannabis use<br>◯ All do not support my medicinal cannabis use<br>(select one option) |
| You have indicated that you have treated your health condition(s) with medicinal cannabis. Have you used medications from the following groups to treat the same health condition(s)? | ☐ Opioids (e.g. morphine, oxycodone, codeine, tramadol, fentanyl, hydromorphone, buprenorphine, methadone)<br>☐ Benzodiazepines or other hypnotics (e.g. diazepam, oxazepam, clonazepam, alprazolam, temazepam, nitrazepam, lorazepam, zopiclone, zolpidem)<br>☐ Antidepressants (e.g. fluoxetine, escitalopram, venlafaxine, amitriptyline, mirtazapine)<br>☐ Antipsychotic medication (e.g. quetiapine, chlorpromazine, olanzipine, amisulpiride)<br>☐ Anticonvulsants (e.g. carbamazepine, valproate, phenytoin, lamotrigine, topiramate, levetiracetam, clonazepam, lacosamide)<br>☐ Gabapentinoids (e.g. Gabapentin, Pregabalin)<br>☐ Analgesics (e.g. ibuprofen, paracetamol)<br>☐ Other<br>☐ I don't use medications to treat my health condition<br>(select all that apply) |
| What other medication have you used to treat the symptoms you now treat with medicinal cannabis? | ______________________________<br>(please list one other medication) |
| You indicated you have used Opioids to treat health conditions that you now use medicinal cannabis to treat. Which of the following best describes how your use of Opioids changed while using medicinal cannabis?<br><br>While using medicinal cannabis my use (either frequency and/or dose) of Opioids: | ◯ Markedly decreased (by 50% or more)<br>◯ Moderately decreased (by 20-50%)<br>◯ More or less stayed the same (no change greater than 20% each way)<br>◯ Moderately increased (by 20-50%)<br>◯ Markedly increased (by 50%)<br>(select one option) |
| You indicated you have used Benzodiazepenes to treat health conditions that you now use medicinal cannabis to treat. Which of the following best describes how your use of Benzodiazepenes changed while using medicinal cannabis?<br><br>While using medicinal cannabis my use (either frequency and/or dose) of Benzodiazepenes: | ☐ Markedly decreased (by 50% or more)<br>☐ Moderately decreased (by 20-50%)<br>☐ More or less stayed the same (no change greater than 20% each way)<br>☐ Moderately increased (by 20-50%)<br>☐ Markedly increased (by 50%)<br>(select one option) |

| | |
|---|---|
| You indicated you have used Antidepressants to treat health conditions that you now use medicinal cannabis to treat. Which of the following best describes how your use of Antidepressants changed while using medicinal cannabis?<br><br>While using medicinal cannabis my use (either frequency and/or dose) of Antidepressants: | ☐ Markedly decreased (by 50% or more)<br>☐ Moderately decreased (by 20-50%)<br>☐ More or less stayed the same (no change greater than 20% each way)<br>☐ Moderately increased (by 20-50%)<br>☐ Markedly increased (by 50%)<br>(select one option) |
| You indicated you have used Antipsychotic medications to treat health conditions that you now use medicinal cannabis to treat. Which of the following best describes how your use of Antipsychotic medications changed while using medicinal cannabis?<br><br>While using medicinal cannabis my use (either frequency and/or dose) of Antipsychotics: | ☐ Markedly decreased (by 50% or more)<br>☐ Moderately decreased (by 20-50%)<br>☐ More or less stayed the same (no change greater than 20% each way)<br>☐ Moderately increased (by 20-50%)<br>☐ Markedly increased (by 50%)<br>(select one option) |
| You indicated you have used Anticonvulsants to treat health conditions that you now use medicinal cannabis to treat. Which of the following best describes how your use of Anticonvulsants medications changed while using medicinal cannabis?<br><br>While using medicinal cannabis my use (either frequency and/or dose) of Anticonvulsants: | ☐ Markedly decreased (by 50% or more)<br>☐ Moderately decreased (by 20-50%)<br>☐ More or less stayed the same (no change greater than 20% each way)<br>☐ Moderately increased (by 20-50%)<br>☐ Markedly increased (by 50%)<br>(select one option) |
| You indicated you have used Gabapentinoids to treat health conditions that you now use medicinal cannabis to treat. Which of the following best describes how your use of Gabapentinoid medications changed while using medicinal cannabis?<br><br>While using medicinal cannabis my use (either frequency and/or dose) of Gabapentinoids: | ☐ Markedly decreased (by 50% or more)<br>☐ Moderately decreased (by 20-50%)<br>☐ More or less stayed the same (no change greater than 20% each way)<br>☐ Moderately increased (by 20-50%)<br>☐ Markedly increased (by 50%)<br>(select one option) |
| You indicated you have used analgesics (e.g. ibuprofen, paracetamol) to treat health conditions that you now use medicinal cannabis to treat. Which of the following best describes how your use of analgesics  changed while using medicinal cannabis?<br><br>While using medicinal cannabis my use (either frequency and/or dose) of analgesics (e.g. ibuprofen, paracetamol): | ☐ Markedly decreased (by 50% or more)<br>☐ Moderately decreased (by 20-50%)<br>☐ More or less stayed the same (no change greater than 20% each way)<br>☐ Moderately increased (by 20-50%)<br>☐ Markedly increased (by 50%)<br>(select one option) |
| How has your use of this other medication has changed since using medicinal cannabis?<br><br>Since using medicinal cannabis my use of this medication has: | ☐ Markedly decreased (by 50% or more)<br>☐ Moderately decreased (by 20-50%)<br>☐ More or less stayed the same (no change greater than 20% each way)<br>☐ Moderately increased (by 20-50%)<br>☐ Markedly increased (by 50%)<br>(select one option) |
| On how many of the last 28 days have you consumed Tobacco? | ____________________________<br>((0-28)) |

Please estimate how many cigarettes (of tobacco) you currently consume per day

__________________________________
(number of cigarettes per day)

---

Since starting medicinal cannabis my use of tobacco has:

- ◯ Markedly decreased (by 50% or more)
- ◯ Moderately decreased (by 20-50%)
- ◯ More or less stayed the same (no change greater than 20% each way)
- ◯ Moderately increased (by 20-50%)
- ◯ Markedly increased (by 50% or more)

(select one option)

---

On how many of the last 28 days did you consume alcohol

__________________________________
((0-28))

---

Please estimate how many standard drinks you consumed per day (on days when you drank).

Guide:
285 ml of beer, 425 of light beer, 100ml of wine, 60ml of fortified wine and 30 ml of spirits, all = 1 standard drink.

A full-strength can or stubby of beer = 1.5 standard drinks.

__________________________________
(In standard drinks. )

---

Since starting use of medicinal cannabis, my use of alcohol has:

- ◯ Markedly decreased (by 50% or more)
- ◯ Moderately decreased (by 20-50%)
- ◯ More or less stayed the same (no change greater than 20% either way)
- ◯ Moderately increased (by 20-50%)
- ◯ Markedly increased (by 50% or more

(select one option)

**SECTION 6**

**SIDE EFFECTS**

**Indicate below whether you have experienced any of the following side effects in the last 12 months when using medicinal cannabis, and, if so, how serious these side effects were.**

**Note: 'Mild and Tolerable' = symptoms that caused only mild impairment of function and do not require specific treatment (e.g. other medications or stopping medicinal cannabis); 'Severe and/or intolerable' = symptoms that caused impairment of function, and/or resulted in either other treatment (e.g. other medications) or resulted in stopping medicinal cannabis use.**

| | Mild and tolerable | Severe and/or intolerable | No |
|---|---|---|---|
| Allergies | ◯ | ◯ | ◯ |
| Anxiety | ◯ | ◯ | ◯ |
| Cannabis hyperemesis | ◯ | ◯ | ◯ |
| Confusion | ◯ | ◯ | ◯ |
| Constipation | ◯ | ◯ | ◯ |
| Decreased appetite | ◯ | ◯ | ◯ |
| Dehydration | ◯ | ◯ | ◯ |
| Depressed mood | ◯ | ◯ | ◯ |
| Diarrhoea | ◯ | ◯ | ◯ |
| Dizziness | ◯ | ◯ | ◯ |
| Drowsiness or sedation | ◯ | ◯ | ◯ |
| Dry mouth | ◯ | ◯ | ◯ |
| Eye irritation (red, sore or itchy eyes) | ◯ | ◯ | ◯ |
| Gastro-intestinal irritation | ◯ | ◯ | ◯ |
| Headaches | ◯ | ◯ | ◯ |
| Increased appetite | ◯ | ◯ | ◯ |
| Lack of energy or fatigue | ◯ | ◯ | ◯ |
| Memory impairment | ◯ | ◯ | ◯ |
| Nasopharyngeal complaints (e.g. sinusitis, pharyngitis) | ◯ | ◯ | ◯ |
| Nausea and/or vomiting | ◯ | ◯ | ◯ |
| Panic attack | ◯ | ◯ | ◯ |
| Paranoia/hallucinations/delusions | ◯ | ◯ | ◯ |
| Racing heart or palpitations | ◯ | ◯ | ◯ |
| Residual bad taste in mouth | ◯ | ◯ | ◯ |

| | | | |
|---|---|---|---|
| Respiratory complaints (e.g. bronchitis, cough) | ○ | ○ | ○ |
| Shaking/tremor/difficulty controlling movements | ○ | ○ | ○ |
| Sleep disturbance | ○ | ○ | ○ |
| Sweating | ○ | ○ | ○ |
| Other | ○ | ○ | ○ |

Please indicate what other type of side effect you have experienced.

__________________________________
(please describe)

## SECTION 7

## CONSEQUENCES OF MEDICINAL CANNABIS USE

**Please indicate below whether you have experienced any of the following IN THE PAST 12 MONTHS**

| | Yes | No |
|---|---|---|
| I often take cannabis in larger amounts or over a longer period of time than I intended to | ○ | ○ |
| I have a persistent desire, or make unsuccessful attempts to cut down or control my cannabis use | ○ | ○ |
| I spend a great deal of time in activities necessary to obtain cannabis, use cannabis, or recover from its effects | ○ | ○ |
| I have cravings, or a strong desire or urge to use cannabis | ○ | ○ |
| My cannabis use results in failure to fulfil my major role obligations at work, school, or home | ○ | ○ |
| I continue to use cannabis despite having persistent or recurrent social or interpersonal problems related to cannabis use (such as criminal charges, ultimatums of abandonment from spouses/partners/friends, and poor productivity | ○ | ○ |
| I have given up or reduced important social, occupational, or recreational activities because of cannabis use | ○ | ○ |
| I recurrently used cannabis in situations in which it is physically hazardous (e.g. driving motor vehicle, operating machinery) | ○ | ○ |

| | | |
|---|---|---|
| I continued to use cannabis even though it causes problems with emotions, mental, or physical health (e.g. cough) | ○ | ○ |
| I needed to use cannabis more than before to get the desired effects or the same amount of cannabis has less effects than before | ○ | ○ |

## SECTION 8

## FINANCIAL AND LEGAL IMPLICATIONS

| | |
|---|---|
| Does the cost of accessing your medicinal cannabis place a significant strain on your finances? | ◯ Yes<br>◯ No |
| Does your medicinal cannabis use make you worry about being arrested or other legal problems? | ◯ Yes<br>◯ No |
| Does your medicinal cannabis use make you worry about the security of your employment? | ◯ Yes<br>◯ No |
| Do you have to do workplace drug testing | ◯ Yes<br>◯ No |
| What should be the legal status of cannabis in Australia? | ◯ Cannabis should be legal for ALL purposes (medical and other)<br>◯ Cannabis should be legal for MEDICAL purposes ONLY<br>◯ Cannabis should be ILLEGAL for ALL reasons<br>◯ Uncertain |

**ATTITUDES TO REGULATION OF MEDICINAL CANNABIS**

**Please indicate the degree to which you agree/ disagree with each of the following statements regarding medicinal cannabis:**

| | Strongly disagree | Disagree | Neither agree or disagree | Agree | Strongly agree |
|---|---|---|---|---|---|
| A person should be able to buy and use medicinal cannabis without approval by a medical practitioner | ◯ | ◯ | ◯ | ◯ | ◯ |
| A person should only be able to legally use medicinal cannabis if a medical professional prescribes it for them | ◯ | ◯ | ◯ | ◯ | ◯ |
| Medicinal cannabis should be considered as part of routine health care in Australia | ◯ | ◯ | ◯ | ◯ | ◯ |
| Medicinal cannabis should meet safety standards (e.g. be of known strength, composition and be free of contaminants) | ◯ | ◯ | ◯ | ◯ | ◯ |
| The cost of medicinal cannabis should be subsidised by the government | ◯ | ◯ | ◯ | ◯ | ◯ |
| The current regulatory framework in Australia for accessing medicinal cannabis works well | ◯ | ◯ | ◯ | ◯ | ◯ |
| The cost of accessing legal medicinal cannabis in Australia is prohibitively expensive | ◯ | ◯ | ◯ | ◯ | ◯ |
| The current model for accessing medicinal cannabis in Australia is 'straightforward' / 'easy to negotiate' for patients | ◯ | ◯ | ◯ | ◯ | ◯ |

Since new rules were introduced around personal use of cannabis in ACT on 31 January 2020, have you started growing your own cannabis?

- ◯ Yes
- ◯ No
- ◯ I'm not aware of any new rules in ACT

Would you be interested in participating in a research study which involves completing a survey on your cannabis use and providing a sample of your cannabis product for chemical analysis for which you will receive individualised results?

- ◯ Yes
- ◯ No

## SECTION 9

## DRIVING

Have you driven a motor vehicle in the past 12 months?
- ◯ Yes
- ◯ No

Have you EVER been convicted of driving under the influence of cannabis?
- ◯ Yes
- ◯ No

After using medicinal cannabis, how long do you typically wait before driving?
- ◯ I drive within one hour of using cannabis
- ◯ I wait 1-3 hours after using cannabis
- ◯ I wait 4-6 hours after using cannabis before driving
- ◯ I wait 7-12 hours after using cannabis before driving
- ◯ I wait 13-24 hours after using cannabis before driving
- ◯ I wait at least 24 hours after using cannabis before driving

In the past 12 months, did you ever drive while under the influence of cannabis (i.e. while you were high)?
- ◯ Yes
- ◯ No

In the past 12 months, how often did you drive while under the influence of cannabis (i.e. while you were high)?
- ◯ Very rarely
- ◯ Rarely
- ◯ Sometimes
- ◯ Often
- ◯ Very often

In the last 12 months, have you undergone road side testing by the police when having used medicinal cannabis?
- ◯ Yes
- ◯ No

Does the presence of roadside drug testing deter you from driving after you have consumed medicinal cannabis?
- ◯ Yes
- ◯ No

Have you been convicted of driving under the influence of cannabis IN THE LAST 12 MONTHS?
- ◯ Yes
- ◯ No

Q 4.19 skipped

Have you EVER been convicted of driving under the influence of cannabis?
- ◯ Yes
- ◯ No

Thinking about how you feel after consuming medicinal cannabis, please indicate to what extent you agree or disagree with the following statements:

I am slower to react to sudden situations
- ◯ Strongly Disagree
- ◯ Disagree
- ◯ Neither agree nor disagree
- ◯ Agree
- ◯ Strongly agree

| | |
|---|---|
| I find it harder to remain focused | ◯ Strongly Disagree<br>◯ Disagree<br>◯ Neither agree nor disagree<br>◯ Agree<br>◯ Strongly agree |
| I tend to drive more carefully | ◯ Strongly Disagree<br>◯ Disagree<br>◯ Neither agree nor disagree<br>◯ Agree<br>◯ Strongly agree |
| I find it harder to stick to the speed limit | ◯ Strongly Disagree<br>◯ Disagree<br>◯ Neither agree nor disagree<br>◯ Agree<br>◯ Strongly agree |
| I tend to leave a larger gap between me and the car ahead | ◯ Strongly Disagree<br>◯ Disagree<br>◯ Neither agree nor disagree<br>◯ Agree<br>◯ Strongly agree |
| I find myself taking more risks | ◯ Strongly Disagree<br>◯ Disagree<br>◯ Neither agree nor disagree<br>◯ Agree<br>◯ Strongly agree |
| I find it harder to drive in a straight line | ◯ Strongly Disagree<br>◯ Disagree<br>◯ Neither agree nor disagree<br>◯ Agree<br>◯ Strongly agree |
| I feel more in control of the vehicle | ◯ Strongly Disagree<br>◯ Disagree<br>◯ Neither agree nor disagree<br>◯ Agree<br>◯ Strongly agree |
| I can accurately assess my driving ability | ◯ Strongly Disagree<br>◯ Disagree<br>◯ Neither agree nor disagree<br>◯ Agree<br>◯ Strongly agree |
| Do you think medicinal cannabis impairs your driving ability? | ◯ Yes<br>◯ No<br>(select one option) |
| Do you think non-medical ('recreational') cannabis impairs your driving ability? | ◯ Yes<br>◯ No |

**SECTION 10**

**GENERAL HEALTH and QUALITY OF LIFE**

**The following three questions assess your psychological health, physical health, and overall quality of life OVER THE LAST 28 DAYS.**

**Please answer on a scale from 0 to 10, with 0 being very poor and 10 being very good.**

| | 0 Poor | 1 | 2 | 3 | 4 | 5 | 6 | 7 | 8 | 9 | 10 Good |
|---|---|---|---|---|---|---|---|---|---|---|---|
| How do you rate your overall PSYCHOLOGICAL health? (consider mood, emotions, and feelings) | ◯ | ◯ | ◯ | ◯ | ◯ | ◯ | ◯ | ◯ | ◯ | ◯ | ◯ |
| How do you rate your overall PHYSICAL health? (consider physical symptoms and/or illness) | ◯ | ◯ | ◯ | ◯ | ◯ | ◯ | ◯ | ◯ | ◯ | ◯ | ◯ |
| How do you rate your overall QUALITY OF LIFE? (e.g. ability to enjoy life, getting on well with family and friends, satisfied with living conditions) | ◯ | ◯ | ◯ | ◯ | ◯ | ◯ | ◯ | ◯ | ◯ | ◯ | ◯ |

Is there anything we have not asked that you would like to add in regards to your experience of medicinal cannabis? (Please do not disclose identifying information such as your name) __________________________________

Please feel free to attach any further comments/feedback on this study
