# Supplementary material for: Medical cannabis use in Australia: consumer experiences from the online cannabis as medicine survey 2020 (CAMS-20)
Source: Harm Reduct J. 2022 Jul 30;19:88. doi: 10.1186/s12954-022-00666-w (PMC9338505; doi:10.1186/s12954-022-00666-w)
Supplement: Supplementary file 2 — Additional file 2: Table S1. Main conditions treated with prescribed and illicit cannabis based on user type. Table S2a. Rates of side effects. Table S2b. Results of ordinal logistic regressions testing differences between prescribed and illicit only users in odds of endorsing more severe side effects. [file 12954_2022_666_MOESM2_ESM.docx]

**Medical cannabis use in Australia: consumer experiences from the online Cannabis as Medicine Survey 2020 (CAMS-20)**

Supplementary Materials

**eTable 1:** Main conditions treated with prescribed and illicit cannabis based on user type

|  |  | **Prescribed Cannabis** | | | | | | **Illicit Cannabis** | | | | | |
| --- | --- | --- | --- | --- | --- | --- | --- | --- | --- | --- | --- | --- | --- |
| **Condition** | **Rank** | **Prescribed Only** (*n*=185) | | **Dual users prescribed** (*n*=349) | | **Total Prescribed** (*n*=534) | | **Dual users illicit** (*n*=319) | | **Illicit Only** (*n*=888) | | **Total Illicit** (*n*=1207) | |
|  |  | **Condition** | ***n* (%)^a^** | **Condition** | ***n* (%)^a^** | **Condition** | ***n* (%)^a^** | **Condition** | ***n* (%)^a^** | **Condition** | ***n* (%)^a^** | **Condition** | ***n* (%)^a^** |
| **Pain** | 1  2  3  4 | **Total**  Arthritis  Neuropathy  Back pain  Fibromyalgia  All others**^b^** | **116 (62.7%)**  30 (16.2%)  20 (10.8%)  17 (9.2%)  15 (8.1%)  34 (18.4%) | **Total**  Back pain  Fibromyalgia  Arthritis  Neuropathy  All others**^b^** | **164 (47.0%)**  49 (14.0%)  26 (7.4%)  25 (7,2%)  21 (6.0%)  43 (12.3%) | **Total**  Back pain  Arthritis  Fibromyalgia  Neuropathy  All others**^b^** | **280 (52.4%)**  66 (12.4%)  55 (10.3%)  41 (7.7%)  41 (7.7%)  77 (14.4%) | **Total**  Back pain  Arthritis  Fibromyalgia  Neuropathy  All others**^b^** | **139 (43.6%)**  43 (13.5%)  22 (6.9%)  20 (6.3%)  20 (6.3%)  34 (10.7%) | **Total**  Back pain  Arthritis  Fibromyalgia  Neuropathy  All others**^b^** | **352 (39.6%)**  96 (10.8%)  92 (10.4%)  48 (5.4%)  35 (3.9%)  44 (5.0%) | **Total**  Back pain  Arthritis  Fibromyalgia  Neuropathy  All others**^b^** | **491 (40.7%)**  139 (11.5%)  114 (9.4%)  68 (5.6%)  55 (4.6%)  115 (9.5%) |
| **Mental Health/**  **Substance Use** | 1  2  3  4 | **Total**  Anxiety  Depression  PTSD | **33 (17.8%)**  22 (11.9%)  6 (3.2%)  5 (2.7%) | **Total**  Anxiety  Depression  PTSD  Addiction  All others**^b^** | **108 (30.9%)**  450 (32.6%)  386 (27.9%)  191 (13.8%)  67 (4.8%)  198 (14.3%) | **Total**  Anxiety  PTSD  Depression  ADHD  All others**^b^** | **141 (26.4%)**  93 (17.4%)  25 (4.7%)  12 (2.2%)  5 (0.9%)  6 (1.1%) | **Total**  Anxiety  PTSD  Depression  ADHD  All others**^b^** | **86 (27.0%)**  54 (16.9%)  13 (4.1%)  10 (3.1%)  2 (0.6%)  7 (2.2%) | **Total**  Anxiety  Depression  PTSD  ADHD  All others**^b^** | **274 (30.9%)**  136 (15.3%)  50 (5.6%)  48 (5.4%)  16 (1.8%)  24 (2.7%) | **Total**  Anxiety  PTSD  Depression  ADHD  All others**^b^** | **360 (29.8%)**  190 (15.7%)  61 (5.1%)  60 (5.0%)  18 (1.5%)  31 (2.6%) |
| **Neurological** | 1  2  3  4 | **Total**  Other**^c^**  MS  Autism  ALS  All others**^b^** | **16 (8.6%)**  9 (4.9%)  3 (1.6%)  2 (1.1%)  1 (0.5%)  1 (0.5%) | **Total**  Other**^c^**  MS  Autism | **20 (5.7%)**  15 (4.3%)  4 (1.1%)  1 (0.3%) | **Total**  Other**^c^**  MS  Autism  ALS  All others**^b^** | **36 (6.7%)**  24 (4.5%)  7 (1.3%)  3 (0.6%)  1 (0.2%)  1 (0.2%) | **Total**  Other**^c^**  Epilepsy  MS  Autism  - | **14 (4.4%)**  6 (1.9%)  4 (1.3%)  3 (0.9%)  1 (0.3%)  - | **Total**  Other**^c^**  Epilepsy  MS  Autism  All others**^b^** | **49 (5.5%)**  31 (3.5%)  8 (0.9%)  5 (0.6%)  3 (0.3%)  2 (0.2%) | **Total**  Other**^c^**  Epilepsy  MS  Autism  All others**^b^** | **63 (5.2%)**  37 (3.1%)  12 (1.0%)  8 (0.7%)  4 (0.3%)  2 (0.2%) |
| **Sleep** | 1  2  3  4 | **Total**  Insomnia  Other**^c^** | **7 (3.8%)**  4 (2.2%)  3 (1.6%) | **Total**  Insomnia  Other**^c^**  Movement  Circadian | **24 (6.9%)**  18 (5.2%)  3 (0.9%)  2 (0.6%)  1 (0.3%) | **Total**  Insomnia  Other**^c^**  Movement  Circadian | **31 (5.8%)**  22 (4.1%)  6 (1.1%)  2 (0.4%)  1 (0.2%) | **Total**  Insomnia  Other**^c^**  Movement  Circadian  All others**^b^** | **47 (14.7%)**  36 (11.3%)  4 (1.3%)  3 (0.9%)  2 (0.6%)  2 (0.6%) | **Total**  Insomnia  Other**^c^**  Movement  Circadian  All others**^b^** | **101 (11.4%)**  67 (7.5%)  12 (1.4%)  8 (0.9%)  6 (0.7%)  8 (0.9%) | **Total**  Insomnia  Other**^c^**  Movement  Circadian  All others**^b^** | **148 (12.3%)**  103 (8.5%)  16 (1.3%)  11 (0.9%)  8 (0.7%)  10 (0.8%) |
| **Gastrointestinal^d^** | 1  2  3  4 | **Total**  Ulc. collitis  Other**^c^** | **3 (1.6%)**  2 (1.1%)  1 (0.5%) | **Total**  Other**^c^**  Crohns  IBS  Ulc. collitis | **8 (2.3%)**  3 (0.9%)  2 (0.6%)  2 (0.6%)  1 (0.3%) | **Total**  Other**^c^**  Ulc. collitis  Crohns  IBS | **11 (2.1%)**  4 (0.7%)  3 (0.6%)  2 (0.4%)  2 (0.4%) | **Total**  Crohn’s  Other**^c^**  IBS  - | **8 (2.5%)**  3 (0.9%)  3 (0.9%)  2 (0.6%)  - | **Total**  IBS  Other**^c^**  Crohn’s  Ulc. collitis | **24 (2.7%)**  9 (1.0%)  7 (0.8%)  6 (0.7%)  2 (0.2%) | **Total**  IBS  Other**^c^**  Crohn’s  Ulc. collitis | **32 (2.6%)**  11 (0.9%)  10 (0.8%)  9 (0.7%)  2 (0.2%) |
| **Cancer** | 1  2  3  4 | **Total**  Blood  Gastro  Other**^c^** | **3 (1.6%)**  1 (0.5%)  1 (0.5%)  1 (0.5%) | **Total**  Other**^c^**  Reproductive  Blood  Breast  All others**^b^** | **8 (2.3%)**  2 (0.6%)  2 (0.6%)  1 (0.3%)  1 (0.3%)  2 (0.6%) | **Total**  Other**^c^**  Blood  Gastro  Reprod  All others | **11 (2.1%)**  3 (0.6%)  2 (0.4%)  2 (0.4%)  2 (0.4%)  2 (0.4%) | **Total**  Breast  Lung  Blood  Other**^c^**  All others**^b^** | **7 (2.2%)**  2 (0.6%)  2 (0.6%)  1 (0.3%)  1 (0.3%)  1 (0.3%) | **Total**  Blood  Breast  Brain  Lung  All others**^b^** | **34 (3.8%)**  7 (0.8%)  7 (0.8%)  6 (0.7%)  4 (0.5%)  10 (1.1%) | **Total**  Breast  Blood  Brain  Lung  All others**^b^** | **41 (3.0%)**  9 (0.7%)  8 (0.7%)  6 (0.5%)  6 (0.5%)  12 (1.0%) |
| **Other** | 1  2  3  4 | **Total**  Other**^c^**  Gyn.  -  - | **7 (3.8%)**  4 (2.2%)  3 (1.6%)  -  - | **Total**  Other**^c^**  Gyn.  Immune  - | **17 (4.9%)**  10 (2.9%)  5 (1.4%)  2 (0.6%)  - | **Total**  Other**^c^**  Gyn.  Immune  - | **24 (4.5%)**  14 (2.6%)  8 (1.5%)  2 (0.4%)  - | **Total**  Other**^c^**  Gyn.  Immune  - | **18 (5.6%)**  9 (2.8%)  6 (1.9%)  3 (0.9%)  - | **Total**  Other**^c^**  Gyn.  Immune  Diabetes  All others**^b^** | **54 (6.1%)**  21 (2.4%)  19 (2.1%)  11 (1.2%)  2 (0.2%)  1 (0.1%) | **Total**  Other**^c^**  Gyn.  Immune  Diabetes  All others**^b^** | **72 (6.4%)**  30 (2.5%)  25 (2.1%)  14 (1.2%)  2 (0.2%)  1 (0.1%) |

*Table ordered by main condition group for all participants who had been prescribed cannabis* *(n=534).* ***a****: percentages displayed represent the proportion each specific condition makes up of the entire group (i.e. 22 respondents using prescribed medical cannabis only reported Anxiety as main condition, which represents 11.9% of the 185 respondents who reported using prescribed only).* ***b:*** *All others’ refers to all the other specific conditions that were listed as a main condition but which were not in the top 4 most commonly.* ***c****: ‘Other’ refers to other conditions that could be classed under the overall main condition but which were not listed in the drop-down list of specific conditions (e.g. other neurological conditions not listed, other sleep conditions not listed). Ulc. Collitis = Ulcerative Collitis; Gyn. = gynaelogical condition; Immune = Auto-Immune condition; Movement = Sleep-related movement disorder; Circadian = Circadian rhythm disorder; MS = Multiple sclerosis; ALS = amyotrophic lateral sclerosis.*

**eTable 2a**: Rates of side effects

|  | **Prescribed** | | | **Illicit only** | | |  |
| --- | --- | --- | --- | --- | --- | --- | --- |
| **Side Effect** | **Mild and Tolerable** | **Severe and Intolerable** | **Any Symptom** | **Mild and Tolerable** | **Severe and Intolerable** | **Total** | **Rank^a^** |
| Allergies, (n=1308) | 14/483 (2.9%) | 2/483 (0.4%) | 16/483 (3.3%) | 32/825 (3.9%) | 3/825 (0.4%) | 35/825 (4.2%) | 26 |
| Anxiety, (n=1309) | 6/483 (1.2%) | 86/483 (17.8%) | 92/483 (19.0%) | 159/826 (19.2%) | 17/826 (2.1%) | 176/826 (21.3%) | 7 |
| Cannabis Hyperemesis, (n=1306) | 9/482 (1.9%) | 1/482 (0.2%) | 10/482 (2.1%) | 12/824 (1.5%) | 0/824 (0.0%) | 12/824 (1.5%) | 29 |
| Confusion, (n=1308) | 2/483 (0.4%) | 59/483 (12.2%) | 61/483 (12.6%) | 3/825 (0.4%) | 86/825 (10.4%) | 89/825 (10.8%) | 13 |
| Constipation, (n=1308) | 4/483 (0.8%) | 24/483 (12.2%) | 28/483 (13.0%) | 3/825 (0.4%) | 39/825 (4.7%) | 42/825 (5.1%) | 21 |
| Decreased Appetite, (n=1308) | 47/483 (9.7%) | 6/483 (1.2%) | 53/483 (10.9%) | 97/825 (11.8%) | 9/825 (1.1%) | 106/825 (12.9%) | 12 |
| Dehydration, (n=1308) | 83/483 (17.2%) | 2/483 (0.4%) | 85/483 (17.6%) | 143/825 (17.3%) | 11/825 (1.3%) | 154/825 (18.6%) | 8 |
| Depressed, (n=1307) | 40/482 (8.3%) | 4/482 (0.8%) | 44/482 (9.1%) | 86/825 (10.4%) | 3/825 (0.4%) | 89/825 (10.8%) | 14 |
| Diaorrhea, (n=1307) | 38/483 (7.9%) | 6/483 (1.2%) | 44/483 (9.1%) | 51/824 (6.2%) | 5/824 (0.6%) | 56/824 (6.8%) | 19 |
| Dizziness, (n=1309) | 80/484 (16.5%) | 4/484 (0.8%) | 84/484 (17.3%) | 92/825 (11.2%) | 7/825 (0.9%) | 99/825 (12.1%) | 10 |
| Drowsiness, (n=1308) | 241/483 (49.9%) | 8/483 (1.7%) | 249/483 (51.6%) | 376/825 (45.6%) | 15/825 (1.8%) | 391/825 (47.4%) | 3 |
| Dry mouth, (n=1308) | 260/483 (53.8%) | 14/483 (2.9%) | 274/483 (56.7%) | 439/825 (53.2%) | 29/825 (3.5%) | 468/825 (56.7%) | 1 |
| Eye irritation, (n=1308) | 118/483 (24.4%) | 2/483 (0.4%) | 120/483 (24.8%) | 203/825 (24.6%) | 11/825 (1.3%) | 214/825 (25.9%) | 5 |
| Fatigue, (n=1308) | 123/483 (25.5%) | 11/483 (2.3%) | 133/483 (27.8%) | 194/825 (23.5%) | 16/825 (1.9%) | 210/825 (25.4%) | 4 |
| Gastrointestinal, (n=1308) | 35/483 (7.3%) | 3/483 (0.6%) | 38/483 (7.9%) | 40/825 (4.9%) | 4/825 (0.5%) | 44/825 (5.4%) | 20 |
| Headaches, (n=1308) | 44/483 (9.1%) | 1/483 (0.2%) | 45/483 (9.3%) | 55/825 (6.7%) | 2/825 (0.2%) | 57/825 (6.9%) | 18 |
| Increased appetite, (n=1309) | 212/483 (43.9%) | 14/483 (2.9%) | 226/483 (46.8%) | 398/826 (48.2%) | 29/826 (3.5%) | 427/826 (51.7%) | 2 |
| Memory problems, (n=1307) | 101/483 (20.9%) | 9/483 (1.9%) | 110/483 (22.8%) | 166/824 (20.2%) | 5/824 (0.6%) | 171/824 (20.8%) | 6 |
| Nasal complaints, (n=1308) | 13/483 (2.7%) | 3/483 (0.6%) | 16/483 (3.3%) | 48/825 (5.8%) | 3/825 (0.4%) | 51/825 (6.2%) | 22 |
| Nausea, (n=1308) | 28/483 (5.8%) | 4/483 (0.8%) | 32/483 (6.6%) | 25/825 (3.0%) | 1/825 (0.1%) | 26/825 (3.1%) | 24 |
| Panic, (n=1308) | 23/483 (4.8%) | 2/483 (0.4%) | 25/483 (5.2%) | 37/825 (4.5%) | 3/825 (0.4%) | 40/825 (4.9%) | 23 |
| Paranoia, (n=1308) | 14/483 (2.9%) | 2/483 (0.4%) | 16/483 (3.3%) | 29/825 (3.5%) | 8/825 (1.0%) | 37/825 (4.0%) | 25 |
| Racing Heart, (n=1308) | 43/483 (8.9%) | 1/483 (0.2%) | 44/483 (9.1%) | 74/825 (9.0%) | 2/825 (0.2%) | 76/825 (9.2%) | 17 |
| Residual bad taste in mouth, (n=1307) | 74/483 (15.3%) | 3/483 (0.6%) | 77/483 (16.1%) | 117/824 (14.2%) | 6/824 (0.7%) | 123/824 (14.9%) | 9 |
| Respiratory complaints, (n=1308) | 41/483 (8.5%) | 0/483 (0.0%) | 41/483 (8.5%) | 123/825 (14.9%) | 12/825 (1.5%) | 135/825 (16.4%) | 11 |
| Sleep disturbance, (n=1308) | 36/482 (7.5%) | 5/483 (1.0%) | 41/483 (8.5%) | 75/825 (9.1%) | 13/825 (1.6%) | 88/825 (10.7%) | 15 |
| Sweating, (n=1307) | 39/483 (8.1%) | 2/483 (0.4%) | 41/483 (8.5%) | 71/824 (8.6%) | 12/824 (1.5%) | 83/824 (10.1%) | 16 |
| Tremors, (n=1307) | 18/482 (3.7%) | 0/482 (0.0%) | 18/482 (3.7%) | 21/825 (2.6%) | 1/825 (0.1%) | 22/825 (2.7%) | 27 |
| Other, (n=1309) | 8/482 (1.7%) | 2/482 (0.4%) | 10/482 (2.1%) | 16/827 (1.9%) | 4/827 (0.5%) | 20/827 (2.4%) | 28 |

*Five most common symptoms highlighted in grey.* ***a:*** *Rank based on the most common symptoms of any severity across both groups*

**eTable 2b**: Results of ordinal logistic regressions testing

differences between prescribed and illicit only users in

odds of endorsing more severe side-effects

|  |  |
| --- | --- |
| **Side Effect** | **OR (95% CI)^a^** |
| Allergies | 1.3 (0.7, 2.4) |
| Anxiety | 1.2 (0.9, 1.5) |
| Cannabis Hyperemesis, (n=1306) | 0.7 (0.3, 1.6) |
| Confusion, (n=1308) | 0.8 (0.6, 1.2) |
| Constipation, (n=1308) | 0.9 (0.5, 1,4) |
| Decreased Appetite, (n=1308) | 1.2 (0.8, 1.7) |
| Dehydration, (n=1308) | 1.1 (0.8, 1.5) |
| Depressed, (n=1307) | 1.2 (0.8, 1.8) |
| Diaorrhea, (n=1307) | 0.7 (0.5, 1.1) |
| Dizziness, (n=1309) | **0.7 (0.5, 0.9)** |
| Drowsiness, (n=1308) | 0.9 (0.7, 1.1) |
| Dry mouth, (n=1308) | 1.0 (0.8, 1.3) |
| Eye irritation, (n=1308) | 1.1 (0.8, 1.4) |
| Fatigue, (n=1308) | 0.9 (0.7, 1.1) |
| Gastrointestinal, (n=1308) | 0.7 (0.4, 1.0) |
| Headaches, (n=1308) | 0.7 (0.5, 1.1) |
| Increased appetite, (n=1309) | 1.2 (1.0, 1.5) |
| Memory problems, (n=1307) | 0.9 (0.7, 1.2) |
| Nasal complaints, (n=1308) | **1.9 (1.1, 3.4)** |
| Nausea, (n=1308) | **0.5 (0.3, 0.8)** |
| Panic, (n=1308) | 0.9 (0.6, 1.6) |
| Paranoia, (n=1308) | 1.4 (0.8, 2.5) |
| Racing Heart, (n=1308) | 1.0 (0.7, 1.5) |
| Residual bad taste in mouth, (n=1307) | 0.9 (0.7, 1.3) |
| Respiratory complaints, (n=1308) | **2.1 (1.5, 3.1)** |
| Sleep disturbance, (n=1308) | 1.3 (0.9, 1.9) |
| Sweating, (n=1307) | 1.2 (0.8, 1.8) |
| Tremors, (n=1307) | 0.7 (0.4, 1.3) |
| Other, (n=1309) | 1.2 (0.5, 2.5) |

*Significant differences in bold.* *Odds ratios >1 indicate the illicit only group were estimated to have greater odds of endorsing more severe symptoms than the prescribed group (vice versa for OR < 1).*
